# Search for polyoma-, herpes-, and bornaviruses in squirrels of the family Sciuridae

**DOI:** 10.1186/s12985-020-01310-4

**Published:** 2020-03-27

**Authors:** Vanessa Schulze, Peter W. W. Lurz, Nicola Ferrari, Claudia Romeo, Michael A. Steele, Shealyn Marino, Maria Vittoria Mazzamuto, Sébastien Calvignac-Spencer, Kore Schlottau, Martin Beer, Rainer G. Ulrich, Bernhard Ehlers

**Affiliations:** 1 Institute of Novel and Emerging Infectious Diseases, Friedrich-Loeffler-Institut, Greifswald - Insel Riems, Germany; 2 Royal (Dick) School of Veterinary Studies and Roslin Institute, University of Edinburgh, Roslin, Scotland UK; 3 Department of Veterinary Medicine, Università degli Studi di Milano, Milan, Italy; 4 Department of Biology, Wilkes University, Wilkes-Barre, PA USA; 5 Department of Theoretical and Applied Sciences, Università degli Studi dell’Insubria, Varese, Italy; 6 P3 “Viral Evolution”, Robert Koch-Institute, Berlin, Germany; 7 Institute of Diagnostic Virology, Friedrich-Loeffler-Institut, Greifswald-Insel Riems, Germany; 8 German Center for Infection Research (DZIF), partner site Hamburg – Lübeck – Borstel – Greifswald-Insel Riems, Greifswald-Insel Riems, Germany; 9 Division 12 ‘Measles, Mumps, Rubella and Viruses Affecting Immunocompromised Patients’, Robert Koch-Institute, Berlin, Germany

**Keywords:** Bornavirus, Polyomavirus, Betaherpesvirus, Gammaherpesvirus, Large T, Small T, VP2, Splicing, Squirrel

## Abstract

**Background:**

Squirrels (family Sciuridae) are globally distributed members of the order Rodentia with wildlife occurrence in indigenous and non-indigenous regions (as invasive species) and frequent presence in zoological gardens and other holdings. Multiple species introductions, strong inter-species competition as well as the recent discovery of a novel zoonotic bornavirus resulted in increased research interest on squirrel pathogens. Therefore we aimed to test a variety of squirrel species for representatives of three virus families.

**Methods:**

Several species of the squirrel subfamilies Sciurinae, Callosciurinae and Xerinae were tested for the presence of polyomaviruses (PyVs; family *Polyomaviridae*) and herpesviruses (HVs; family *Herpesviridae*), using generic nested polymerase chain reaction (PCR) with specificity for the PyV VP1 gene and the HV DNA polymerase (DPOL) gene, respectively. Selected animals were tested for the presence of bornaviruses (family *Bornaviridae*), using both a broad-range orthobornavirus- and a variegated squirrel bornavirus 1 (VSBV-1)-specific reverse transcription-quantitative PCR (RT-qPCR).

**Results:**

In addition to previously detected bornavirus RNA-positive squirrels no more animals tested positive in this study, but four novel PyVs, four novel betaherpesviruses (BHVs) and six novel gammaherpesviruses (GHVs) were identified. For three PyVs, complete genomes could be amplified with long-distance PCR (LD-PCR). Splice sites of the PyV genomes were predicted in silico for large T antigen, small T antigen, and VP2 coding sequences, and experimentally confirmed in Vero and NIH/3T3 cells. Attempts to extend the HV DPOL sequences in upstream direction resulted in contiguous sequences of around 3.3 kilobase pairs for one BHV and two GHVs. Phylogenetic analysis allocated the novel squirrel PyVs to the genera *Alpha*- and *Betapolyomavirus*, the BHVs to the genus *Muromegalovirus*, and the GHVs to the genera *Rhadinovirus* and *Macavirus*.

**Conclusions:**

This is the first report on molecular identification and sequence characterization of PyVs and HVs and the detection of bornavirus coinfections with PyVs or HVs in two squirrel species. Multiple detection of PyVs and HVs in certain squirrel species exclusively indicate their potential host association to a single squirrel species. The novel PyVs and HVs might serve for a better understanding of virus evolution in invading host species in the future.

## Background

Squirrels (family Sciuridae) are members of the order Rodentia, and with the exception of Antarctica, distributed globally on all continents. They occur both in the Old and in the New world, and squirrel species diversity is particularly high in Africa and Asia [1]. Squirrels are generally distinguished as ground, flying (gliding) or tree squirrels. With respect to the latter, the Eurasian red squirrel (*Sciurus vulgaris*) is the dominant squirrel species across the Palaearctic [2, 3]. In contrast to the North American red or pine squirrel (*Tamiasciurus hudsonicus*) which is a boreal coniferous species [1], the Eurasian red squirrel originally occupied all available forest habitats in the absence of other tree squirrels across most of its range.

The Eastern grey squirrel (*Sciurus carolinensis*) is a broadleaf specialist and was originally distributed in the eastern deciduous forests of Northern America [4]. However, the species has been translocated repeatedly within North America and globally to Europe, South Africa and Australia (e.g. see [4, 5]). The multiple introductions and subsequent translocations had devastating consequences for local Eurasian red squirrel populations in deciduous and mixed forest landscapes in Great Britain, Ireland and Italy leading to large-scale declines as a result of competition for resources and introduced pathogens [6–8]. With regard to pathogens, the replacement of native Eurasian red squirrels by the Eastern grey squirrels in the British Isles is highly accelerated by a squirrelpox virus (e.g. [9]). However, the resulting research interest on pathogens also resulted in the identification of other potential disease threats to red squirrels such as leprosy bacilli [10] and adenovirus [11–13]. Although squirrel adenovirus has been known for many years, it was only recently that the complete genome of squirrel adenovirus 1 (SqAdV-1) was determined and found to indicate a close relationship between British and Continental European red squirrel populations [11, 13].

Prevost’s squirrels (*Callosciurus prevostii*) and Pallas’s squirrels (*Callosciurus erythraeus*) belong to the subfamily Callosciurinae. Both species are native to South-East Asia and Pallas’s squirrels were introduced to Italy, France, Belgium and the Netherlands. They typically represent non-native, invasive tree squirrel species that arrived in Europe via pet trade [14–16]. The variegated squirrel (*Sciurus variegatoides*), a species of dry tropical forests in Central America, is another tree squirrel that appears popular among squirrel breeders [17], perhaps due to its great variability in coat colour [18]. Whilst there has been some progress in recognition of potential threats to wildlife and people from introduced species (e.g. [19, 20]), little information is available about viral and bacterial pathogens and parasites in squirrels and the role of squirrels in population dynamics, competitive interactions or as reservoirs for zoonotic diseases.

Recently a novel zoonotic bornavirus, variegated squirrel bornavirus 1 (VSBV-1), was detected in five different squirrel species from private holdings and zoos, including *Sciurus variegatoides* and *Callosciurus prevostii* and is associated with cases of fatal encephalitis of their breeders and care takers [17, 21–23]. Bornaviruses (family *Bornaviridae*) are enveloped spherically structured viruses with a single-stranded RNA genome of negative polarity (size around 8.9 kilobases (kb)). Bornaviruses have been identified in a wide range of hosts, e.g. mammals, birds and reptiles [24]. Members of the genus *Orthobornavirus* infecting mammals are assigned to two species: *Mammalian 1 orthobornavirus* (Borna Disease Virus 1 and 2; BoDV-1 and BoDV-2) and *Mammalian 2 orthobornavirus* (VSBV-1). These viruses have pathogenic potential for humans and other mammals. BoDV-1 is the causative agent of Borna disease, an often fatal neurologic condition of horses, sheep and other domestic mammals. Recently, its zoonotic potential has been demonstrated by molecular and immunohistochemical detection of several BoDV-1-induced fatal encephalitis cases in humans [25–28].

Polyomaviruses (PyVs; family *Polyomaviridae*) and herpesviruses (HVs; family *Herpesviridae*) both comprise a plethora of viruses many of which are pathogenic for humans and animals. In members of some mammalian orders (e.g. primates, artiodactyls or rodents) many PyVs and HVs have been identified, some of which are well studied [29–33]. Despite this knowledge, information on PyVs and HVs is still scarce for many mammalian families. This holds particularly true for the family Sciuridae. To our knowledge, there is no report describing the occurrence of PyVs in members of the Sciuridae. From the 1980s there exist descriptions of HVs in a thirteen-lined ground squirrel (*Ictidomys tridecemlineatus*) [34], and *Citellus* spp. (now *Spermophilus*) [35, 36] but these reports are based only on electron microscopy and cytopathogenic effects in infected cell cultures, which were assumed to be typical for HVs, despite the lack of sequence data.

PyVs are small non-enveloped viruses with an icosahedral capsid and a circular double-stranded DNA genome that consists of approximately 5 kilobase pairs (kbp). The family *Polyomaviridae* comprises around 100 members, including 14 that infect humans. PyVs have been found in many hosts, including humans, nonhuman primates, rodents, cattle, bats, birds, and fish [31]. PyVs cause subclinical infections, as well as acute systemic diseases, the latter mainly in immune-compromised individuals. Some PyVs have transforming activity in vitro and reveal tumorigenic capacity in laboratory animals. Merkel cell polyomavirus (MCPyV) is the first human PyV that is associated with a tumor in humans [37, 38].

The genomic organization of mammalian PyVs comprises three regions: The early transcriptional region, the late transcriptional region and the non-coding control region (NCCR). The early region encodes regulatory proteins, including large T-antigen (LTAg) and small T-antigen (STAg). The counter clock-wise oriented late region encodes the structural proteins VP1, VP2, and VP3. Early and late regions are separated by the NCCR, which controls DNA replication and transcription from the early and late promoters [39]. LTAg and STAg are involved in viral transcription and replication. LTAg induces the synthesis phase of cells and can cause an abnormal stimulation of the cell cycle and tumor formation [31, 39, 40]. Mammalian PyVs are assigned to three distinct genera within the family *Polyomaviridae: Alpha-, Beta-,* and *Deltapolyomavirus* [41].

HVs are a family of large, enveloped viruses with a double-stranded DNA genome (length: 110–295 kbp) that infect many vertebrates, including humans and nonhuman primates [29]. Mammalian HVs are divided into three distinct subfamilies within family *Herpesviridae*: *Alphaherpesvirinae, Betaherpesvirinae* (BHVs) and *Gammaherpesvirinae* (GHVs)*.* All HVs share the capacity to establish a state of latency resulting in lifelong association with the infected host. After reactivation, an infectious virus is produced and spreads to other susceptible individuals [29, 42]. Herpesviruses cause a variety of diseases in humans and animals, including some cancers.

As bornaviruses have so far only been identified in five of the > 280 sciurid species, and sciurid PyVs and HVs are not yet known, we sought to improve our knowledge on such viruses in the Sciuridae family and performed a molecular survey in squirrels of different species and subfamilies. Spleen and lung samples of 238 animals from five countries (Canada, USA, Italy, UK and Germany) were tested with generic nested PCRs [43–49] for the identification of PyVs and HVs. Brain samples from 126 of these animals originating from four countries (Canada, USA, Italy and UK) were analyzed with broad-range orthobornavirus RT-qPCR for the generic detection of orthobornaviruses and a VSBV-1-specific reverse transcription-quantitative polymerase chain reaction (RT-qPCR). In case of successful PyV sequence detection, we aimed at generating complete genome sequences, and in case of HV sequence detection, we wanted to amplify and sequence a genome segment, which comprises around 3.4 kbp and extends from the glycoprotein B (gB) gene to the DNA polymerase (DPOL) gene. This approach led to the discovery of four PyVs and 10 HVs (four BHVs and six GHVs).

## Methods

### Sample collection and nucleic acid preparation

Squirrels (*n* = 126) of five species belonging to the family Sciuridae were collected in four different countries (Canada, USA, Italy and UK), dissected according to standard protocols and brain, lung and spleen samples of these animals were available for screening for all three viruses. In addition, lung and spleen samples from another 112 squirrels belonging to another three species were available from previous bornavirus studies [17, 21, 22]. Thus, a total of 361 organ samples (243 spleen samples and 118 lung samples) were available for PyV and HV analyses (Tables 1, 2 and 3).

**Table 1:** Bornavirus reverse transcription - quantitative polymerase chain reaction (RT-qPCR) analysis of squirrels from wildlife and holdings

Host taxonomic name (subfamily, species)	Host common name	n positive/ total n tested in this study	RT-qPCR positive/ total n tested in previous studies [17, 21, 22]
**Sciurinae**			
*Sciurus carolinensis*	Eastern grey squirrel	0/77^a^	0/11^a^
*Sciurus variegatoides*	Variegated squirrel		7/7^b^
*Sciurus vulgaris*	Eurasian red squirrel		0/77^a^
*Tamiasciurus hudsonicus*	American red squirrel	0/1^a^	
***Xerinae***			
*Urocitellus richardsonii*	Richardson’s ground squirrel	0/11^a^	
*Tamias striatus*	Eastern chipmunk	0/2^a^	
***Callosciurinae***			
*Callosciurus erythraeus*	Pallas’s squirrel	0/35^a^	
*Callosciurus prevostii*	Prevost’s squirrel		10/17^b^
**total**		**0/126**	**17/112**

^a^all individuals originated from wildlife

^b^all individuals originated from holdings

**Table 2:** Geographic origin of identified polyomaviruses in members of the Sciuridae and detailed PCR results

Host taxonomic name (subfamily, species)	Host common name	Origin^a^	Polyomavirus	Organs tested with PCRs	n tested squirrels	n tested samples	n samples positive in generic nested PCR	n samples positive in virus specific nested PCR	n animals positive in generic or specific nested PCR
**Sciurinae**									
*Sciurus carolinensis*	Eastern grey squirrel	Penicuik, Scotland	ScarPyV1	lung, spleen	6	11	1	8	6
		Anwoth, Scotland		lung, spleen	3	6	0	0	0
		Kirkbright, Scotland		lung, spleen	4	8	0	0	0
		Dumfries, Scotland	ScarPyV1	spleen	13	13	n. d.^**b**^	9	9
		Borders Region, Scotland	ScarPyV1	spleen	13	13	n. d.	4	4
		Brampton, England	ScarPyV1	spleen	10	10	n. d.	9	9
		Pennsylvania, USA	ScarPyV1	lung	5	5	0	2	2
		Piedmont Region, Italy	ScarPyV1	lung, spleen	30	45	0	1	1
		Lomardy Region, Italy		lung, spleen	4	7	0	0	0
***subtotal***					**88**	**118**	**1**	**33**	**31 (35.2%)**
*Sciurus vulgaris*	Eurasian red squirrel	Scotland (road mortality)		lung, spleen	7	12	0	- ^**c**^	0
		Isle of Arran, Scotland		lung, spleen	12	13	0	–	0
		Neustadt, Germany		lung, spleen	1	2	0	–	0
		Munich, Germany		lung, spleen	16	22	0	–	0
		Waiblingen, Germany		lung, spleen	2	4	0	–	0
		Heidelberg, Germany		spleen	1	1	0	–	0
		Bad König, Germany		lung, spleen	2	4	0	–	0
		Dresden, Germany		lung, spleen	1	2	0	–	0
		Wentorf, Germany		lung, spleen	1	2	0	–	0
		Billigheim, Germany		lung, spleen	1	2	0	–	0
		Rödermark, Germany		lung, spleen	6	12	0	–	0
		Leutenbach, Germany		lung, spleen	1	2	0	–	0
		Bonn, Germany		lung	1	1	0	–	0
		Bad Nauheim, Germany		lung	1	1	0	–	0
		Oberhausen, Germany		lung	1	1	0	–	0
		Berlin, Germany		lung, spleen	4	8	0	–	0
		Stuttgart, Germany		lung, spleen	6	12	0	–	0
		Görlitz, Germany		lung, spleen	6	12	0	–	0
		Sternenfels, Germany		lung, spleen	7	13	0	–	0
***subtotal***					**77**	**126**	**0**		**0**
*Sciurus variegatoides*	Variegated squirrel	Germany	SvarPyV1	spleen	7	7	1	–	1 **(14.3%)**
*Tamiasciurus hudsonicus*	American red squirrel	Pennsylvania, USA		lung	1	1	0	–	0
***Xerinae***									
*Urocitellus richardsonii*	Richardson’s ground squirrel	Winnipeg, Canada		spleen	11	11	0	–	0
*Tamias striatus*	Eastern Chipmunk	Pennsylvania, USA		lung, spleen	2	2	0	–	0
***Callosciurinae***									
*Callosciurus erythraeus*	Pallas’s squirrel	Lombardy Region, Italy	CeryPyV1	lung, spleen	35	44	8	18	15 **(42.9%)**
*Callosciurus prevostii*	Prevost’s squirrel	Germany	CprePyV1	spleen	17	17	3	4	4 **(23.5%)**
**total**					**238**	**326**	**13**	**55**	**51**

^a^all animals originated from wildlife, except variegated squirrels and Prevost’s squirrels originating from holdings in Germany

^b^*n. d.* not done

^c^- = specific nested PCR was not possible because no polyomavirus was detected in generic PCR or specific nested primers could not be designed because LD-PCR was unsuccessful

**Table 3:** Geographic origin of identified herpesviruses in members of the Sciuridae and detailed PCR results

Host taxonomic name (subfamily, species)	Host common name	Origin^a^	Herpesvirus	Organs tested with PCRs	n tested squirrels	n tested samples	n samples positive in generic PCR	n samples positive in virus specific nested PCR	n animals positive in generic or specific nested PCR
**Sciurinae**									
*Sciurus carolinensis*	Eastern grey squirrel	Penicuik, Scotland	ScarGHV1	lung, spleen	6	11	8	n. d.^**b**^	5
		Anwoth, Scotland		lung, spleen	3	6	0	- ^**c**^	0
		Kirkbright, Scotland	ScarGHV1	lung, spleen	4	8	2	n. d.	1
		Dumfries, Scotland	ScarGHV1	spleen	13	13	2	n. d.	2
			ScarGHV2	spleen			1	n. d.	1
		Borders Region, Scotland	ScarGHV1	spleen	13	13	3	n. d.	3
		Brampton, England	ScarGHV1	spleen	10	10	9	n. d.	9
		Pennsylvania, USA	ScarGHV1	lung	5	5	3	n. d.	3
			ScarGHV2	lung			1	0	1
		Piedmont Region, Italy	ScarBHV1	lung, spleen	30	45	2	4	4
		Lombardy Region, Italy	ScarBHV1	lung, spleen	4	7	0	1	1
***subtotal***		**ScarGHV1**			**88**	**118**	**29**	**n. d.**	**28 (31.8%)**
		**ScarGHV2**			**88**	**118**	**2**	**0**	**2 (2.3%)**
		**ScarBHV1**			**88**	**118**	**2**	**5**	**5 (5.7%)**
*Sciurus vulgaris*	Eurasian red squirrel	Scotland (Scotland (road mortality))	SvulBHV1	lung, spleen	7	12	9	n. d.	6
		Isle of Arran, Scotland	SvulBHV1	lung, spleen	12	13	7	n. d.	7
		Neustadt, Germany		lung, spleen	1	2	0	–	0
		Munich, Germany	SvulBHV1	lung, spleen	16	22	9	n. d.	7
		Waiblingen, Germany		lung, spleen	2	4	0	–	0
		Heidelberg, Germany		spleen	1	1	0	–	0
		Bad König, Germany		lung, spleen	2	4	0	–	0
		Dresden, Germany		lung, spleen	1	2	0	–	0
		Wentorf, Germany	SvulBHV1	lung, spleen	1	2	1	n. d.	1
		Billigheim, Germany		lung, spleen	1	2	0	–	0
		Rödermark, Germany	SvulBHV1	lung, spleen	6	12	3	n. d.	2
		Leutenbach, Germany	SvulBHV1	lung, spleen	1	2	1	n. d.	1
		Bonn, Germany	SvulBHV1	lung	1	1	1	n. d.	1
		Bad Nauheim, Germany		lung	1	1	0	–	0
		Oberhausen, Germany		lung	1	1	0	–	0
		Berlin, Germany	SvulBHV1	lung, spleen	4	8	3	n. d.	2
		Stuttgart, Germany	SvulBHV1	lung, spleen	6	12	1	n. d.	1
		Görlitz, Germany	SvulBHV1	lung, spleen	6	12	2	n. d.	2
		Sternenfels, Germany	SvulBHV1	lung, spleen	7	13	2	n. d.	1
***subtotal***					**77**	**126**	**39**		**31 (40.3%)**
*Sciurus variegatoides*	Variegated squirrel	Germany		spleen	7	7	0	–	0
*Tamiasciurus hudsonicus*	American red squirrel	Pennsylvania, USA		lung	1	1	0	–	0
**Xerinae**									
*Urocitellus richardsonii*	Richardson’s ground squirrel	Winnipeg, Canada	UricGHV1	spleen	11	11	10	n. d.	10 **(90.9%)**
*Tamias striatus*	Eastern Chipmunk	Pennsylvania, USA	TstrGHV1	lung, spleen	2	2	2	2	2 **(100.0%)**
**Callosciurinae**									
*Callosciurus erythraeus*	Pallas’s squirrel	Lombardy Region, Italy	CeryBHV1	lung, spleen	35 35	44	18	11	23 **(65.7%)**
			CeryGHV1	lung, spleen		44	2	5	6 **(14.3%)**
*Callosciurus prevostii*	Prevost’s squirrel	Germany	CpreBHV1	spleen	17 17	17	1	1	1 **(5.9%)**
			CpreGHV1	spleen		17	1	1	1 **(5.9%)**
**total**					**238**	**326**	**104**	**27**	**108**

^a^all animals originated from wildlife, except variegated squirrels and Prevost’s squirrels originating from holdings in Germany

^b^*n. d.* not done

^c^- = specific nested PCR was not possible because no herpesvirus was detected in generic PCR

RNA extraction was performed using the KingFisher™ Flex Purification System (Thermo Fisher) in combination with the Nucleo Mag Vet Kit (Macherey Nagel) following the instructions of the manufacturer. DNA extraction was performed using EURx GeneMatrix Tissue DNA Purification Kit (Roboklon), Qiagen DNeasy Blood & Tissue-Kit (Qiagen) and the KingFisher™ Flex Purification System (Thermo Fisher) in combination with the Nucleo Mag Vet Kit (Macherey Nagel). DNA preparations were stored at − 20 °C and RNA preparations at − 80 °C. During the nucleic extraction processes negative controls were carried along to monitor for potential contaminations. Morphological species identification was confirmed for all samples by cytochrome *b* PCR and sequencing according to a previously described protocol ( [50], data not shown). Prior to nucleic acid extraction no attempts concerning virus isolation in cell culture were done.

### Bornavirus screening of squirrel brain samples

Brain samples of the newly dissected 126 squirrels were screened with broad-range and VSBV-1 specific RT-qPCRs (Additional file 1) as described previously [17, 28]. The other animals (*n* = 112) have already been investigated before [17, 21, 22].

### Identification of polyomaviruses and herpesviruses with generic PCR assays

For identification of PyVs and HVs generic nested PCRs were performed, that broadly detect a partial VP1 coding sequence (CDS) of PyVs (Additional files 1 and 2) or a fragment of the DPOL gene of HVs (Open reading frame 09 (ORF09) of GHV; ORF UL54 of BHV) (Additional file 1 and Additional file 3) in the second PCR round. Both nested PCRs were performed as carried out previously [46, 48].

### Amplification of gB sequences of betaherpesviruses and gammaherpesviruses with generic PCR

For amplification of gB sequences of BHVs (ORF UL55) and GHVs (ORF08), we used subfamily-specific nested primer sets (Additional file 1) essentially as described previously [51, 52]. The scheme of multi-level PCR analysis used for squirrel BHVs and GHVs is shown in Additional file 3.

### Nested long-distance PCR with virus-specific primers

For all PyVs, specific nested primers (Additional file 1) were selected tail-to-tail from the sequences amplified with generic PCR. They were used for the amplification and sequencing of the remaining parts of the circular genomes (approximately 5 kbp). For all HVs, for which both gB and DPOL sequences could be amplified, nested primers (Additional file 1) were selected that were specific for each virus and used in long-distance PCR (LD-PCR) for the amplification and sequencing of the sequence that spans the gap between the partial gB and DPOL sequences. LD-PCR was performed with the TaKaRa-Ex PCR system (Takara Bio Inc.), according to the manufacturer’s instructions. After the first PCR round, a 2 μl aliquot of the reaction mix was used as template in the second-round reaction.

### Hemi-nested herpesvirus DPOL PCR

In cases where only the generic HV PCR was successful (and not gB PCR and/or LD-PCR), the short DPOL sequence was extended in upstream direction with hemi-nested PCR, using the outer sense primer (285 DFA) of the generic HV PCR and two virus-specific antisense primers for amplification of approximately 480 base pairs (bp) (Additional file 1). The hemi-nested DPOL PCR was carried out as described above for generic HV PCR.

Based on these extended sequences we were able to design again virus-specific primers that were used for re-screening of all samples of the respective species.

### Nested PCR with polyomavirus-specific primers

For all novel PyVs, for which full genomes could be assembled, nested primer sets (Additional file 1) were selected. They were used for amplification of sequences of approximately 800 bp that encompass the short overlap between the sequences generated from the generic PCR fragments and the LD-PCR fragments of the respective PyV genome. PCR was performed in a total volume of 25 μl with 0.4 μl (2 units) Applied Biosystems AmpliTaq Gold DNA Polymerase (Thermo Fisher), 25 pmol of each primer, 200 μM dideoxynucleoside triphosphates (dNTPs), 2 mM MgCl_2_, 5% dimethyl sulfoxide (DMSO) and 250 ng of sample DNA as template. A thermocycler from Biometra was used under the following cycling conditions: activation of the polymerase at 95 °C for 12 min and 45 cycles of denaturation at 95 °C for 30 s, annealing at 61 °C for 30 s, and elongation at 72 °C for 5 min, followed by a final extension step at 72 °C for 30 min. For the second PCR round, a 1 μl aliquot of the first-round reaction mix was used as a template. The primers were also used for more sensitive re-screening all samples of the respective host species.

### Nested PCR with herpesvirus-specific primers

For all novel HVs, for which extended sequences could be determined, nested primer sets were selected for re-screening all samples of the respective host species (Additional file 1). They were used as described above for specific PyV amplification (annealing temperatures listed in Additional file 1).

### RT-PCR and PCR controls

For each round of all nested PCRs and the RT-qPCR of our screening, no-template controls (PCR-grade H_2_O) and extraction controls were carried along to detect any possible contaminations. These analyses were negative for all PCRs and RT-qPCRs. As positive PCR controls, DNA extracts of samples were used that tested positive for BHVs, GHVs or PyVs in previous studies.

### PCR product purification and sequencing

All PCR products were purified with MSB® Spin PCRapace (Stratec), according to the manufacturer’s instruction and directly sequenced using the BigDye Terminator v3.1 system (Life Technologies) on an Applied Biosystems 3500xL DX Genetic Analyzer (Thermo Fisher). The LD-PCR products were sequenced by a classical primer walking strategy (primers not listed).

### Synthesis of polyomavirus early and late region

Early or late region plus flanking sequences (approximately 3.1 kbp) of *Sciurus carolinensis* polyomavirus 1 (ScarPyV1; GenBank accession number MK671101) were commercially synthesized and delivered as recombinant plasmids (Biomatik). They were named pScarPyV1early and pScarPyV1late and transformed into competent *Escherichia coli* DH5 alpha cells (Thermo Fisher). Plasmid DNA was extracted with Invisorb® Spin Plasmid Mini Two (Stratec) according to the manufacturer’s instruction.

### Cell lines

Vero cells C1008 (monkey kidney cells; European Collection of Authenticated Cell Cultures (ECACC) # 85020206) were cultured in standard high-glucose Dulbecco’s minimal Eagle medium (DMEM, Thermo Fisher) containing 10% fetal calf serum (FCS) (PAN Biotech) and 1% penicillin/streptomycin (Thermo Fisher). For NIH/3T3 cells (mouse embryo fibroblast cells; American Tissue Culture Collection, ATCC®, CRL-1658™) the same medium was used, except that 5% FCS (PAN Biotech) was added. Both cell lines were cultivated at 37 °C and 5% CO_2_. DNA extracts of cell aliquots were tested with PCR for absence of mycoplasma contamination [53]. Primers are listed in Additional file 1.

### Transfection of cells

As described previously [54], cells were transfected with 1 μg DNA of plasmid pScarPyV1early or plasmid pScarPyV1late, using X-tremeGENE HP DNA Transfection Reagent (Roche Applied Biosciences). Before transfection, Vero cells were seeded in a volume of 500 μl cell culture medium in tissue culture plates with 24 wells (Sarstedt). For NIH/3T3 cells, Cell+ plates with 24 wells and a special Cell+ growth surface for sensitive adherent cells (Sarstedt) were used. Transfection procedures were performed 24 h after seeding according to the manufacturer’s instructions.

### RNA extraction and cDNA synthesis

Total RNA was isolated on 0, 1, 2.5, and 6 days post transfection of recombinant plasmid DNA using the NucleoSpin® RNA-Kit (Macherey Nagel) according to the manufacturer’s instructions. DNA was removed by an additional Turbo DNA-free DNase treatment (Thermo Fisher). RNA concentrations were determined with the NanoDrop 8000 device (Thermo Fisher) at 260 nm. Synthesis of cDNA was carried out with 500 ng RNA using SuperScript II Reverse Transcriptase (Thermo Fisher) and oligo(dT)16 primers (Roche Applied Bioscience).

### PCR for identification of introns

To identify introns in the early and late region of ScarPyV1, PCR was performed with cDNA as a template, using primers specific for ScarPyV1 that bind in flanking regions of predicted introns (Additional file 1). PCR was performed with 2.5 μl cDNA in a total volume of 25 μl with 0.2 μl (1 unit) Applied Biosystems AmpliTaq Gold DNA Polymerase (Thermo Fisher), 1 μM of each primer, 200 μM dNTP PCR Mix (Metabion), 2 mM MgCl_2_ and 5% DMSO (Merck). All PCR products were purified with MSB® Spin PCRapace (Stratec), according to the manufacturer’s instructions. Sequencing reactions were performed as described above.

### Bioinformatics and phylogenetic analysis

For the datasets we selected reference viral genomes representing all currently recognized species in the family/subfamily considered as well as additional viruses whose genomes represented distinct viral lineages discussed in the literature but still not integrated into the official taxonomy (sensu International Committee on Taxonomy of Viruses (ICTV)). For PyVs, this represented 109 viruses; for BHVs, 21 viruses; and for GHVs, 39 viruses. We extracted the LTAg and VP1 (PyV) or DPOL and gB (BHV and GHV) coding sequences from these genomes as well as from the novel viruses we identified in squirrels using Geneious v11.1.5 [55]. For each coding sequence, sequences were translated into amino acid sequences and aligned using Muscle [56] as implemented in Seaview v4 [57]. Conserved amino acid blocks were then selected using Gblocks as implemented in Seaview, using options for a less stringent selection: allow smaller final blocks, allow gap positions within the final blocks and allow less strict flanking positions [58]. The final amino acid sequence alignments comprised 260 (VP1), 517 (LTAg), 628 (DPOL BHV), 298 (gB BHV), 622 (DPOL GHV) and 282 (gB GHV) positions, respectively.

For phylogenetic analyses of all datasets we first ran Maximum-Likelihood (ML) analyses using PhyML v3 with smart model selection (PhyML-SMS) using the Bayesian information criterion and a tree search using subtree pruning and regrafting [57, 59, 60]. Branch robustness was estimated using Shimodaira-Hasegawa-like approximate likelihood ratio tests (SH-like aLRT) [61]. The PyV and GHV ML trees were rooted with TempEst v1.5 by minimizing the variance of root-to-tip distances [62]; the BHV ML trees were rooted using roseolovirus outgroups. We then ran Bayesian Markov chain Monte Carlo (BMCMC) runs using BEAST v1.10.4 [63]. For each alignment, we used the amino acid substitution model identified by PhyML-SMS, an uncorrelated relaxed clock (lognormal) model and a speciation model (birth-death) as a tree prior. The output of multiple BMCMC runs was examined for convergence and appropriate sampling of the posterior using Tracer v1.7.1 [64], before being merged using LogCombiner v1.10.4 (distributed with BEAST). The maximum clade credibility (MCC) tree was identified from the posterior set of trees (PST) and annotated with TreeAnnotator v1.10.4 (also distributed with BEAST). Branch robustness was estimated based on their posterior probability in the PST.

## Results

### Bornavirus analysis

In previous studies, brain and oral swab samples of 112 squirrels belonging to various species were investigated with a broad-range orthobornavirus RT-qPCR and VSBV-1-specific RT-qPCR. VSBV-1-RNA was detected in brain and/or oral swab samples of 7/7 individuals from holdings of variegated squirrels, in samples of 10/17 Prevost's squirrels and a few other species [17, 21, 22], but not in any brain samples of the 77 tested Eurasian red squirrels and in any of the 11 Eastern grey squirrels [17, 21, 22]. Here we investigated a total of 126 brain samples from wildlife-derived squirrels from four countries, but did not find any positive specimens (Table 1).

### Identification of novel polyomaviruses and herpesviruses

To identify PyVs and HVs in squirrels, we analyzed 238 squirrels (spleen, *n* = 208; lung, *n* = 118) of eight different species (Tables 2 and 3). First we screened with generic nested PCR that targets a short fragment of the major capsid VP1 CDS of mammalian PyVs (Additional file 1 and Additional file 2) [43, 47–49] and generic nested PCR with specificity for the highly conserved DPOL gene of mammalian HVs (Additional file 1 and Additional file 3) [44–46]. Products of expected length were sequenced and sequences analyzed using Nucleotide Basic Local Alignment Search Tool (BLASTn). Thirteen samples of twelve animals were positive for yet unknown PyVs (Table 2) and 104 samples of 89 animals were positive for yet unknown HVs (Table 3) as revealed by BLASTn analysis (data not shown). In total, four novel PyVs, and 10 novel HVs (four BHVs and six GHVs) were identified and tentatively named: *Sciurus carolinensis* polyomavirus 1 (ScarPyV1), *Sciurus carolinensis* betaherpesvirus 1 (ScarBHV1), *Sciurus carolinensis* gammaherpesvirus 1 and 2 (ScarGHV1 and ScarGHV2), *Sciurus variegatoides* polyomavirus 1 (SvarPyV1), *Sciurus vulgaris* betaherpesvirus 1 (SvulBHV1), *Callosciurus erythraeus* polyomavirus 1 (CeryPyV1), *Callosciurus erythraeus* betaherpesvirus 1 (CeryBHV1), *Callosciurus erythraeus* gammaherpesvirus 1 (CeryGHV1), *Callosciurus prevostii* polyomavirus 1 (CprePyV1), *Callosciurus prevostii* betaherpesvirus 1 (CpreBHV1), *Callosciurus prevostii* gammaherpesvirus 1 (CpreGHV1), *Urocitellus richardsonii* gammaherpesvirus 1 (UricGHV1) and *Tamias striatus* gammaherpesvirus 1 (TstrGHV1).

In PyV detection with generic PCR, one of 118 spleen samples and one of 88 lung samples from Eastern grey squirrels (*Sciurus carolinensis*) were positive for ScarPyV1, 1/7 spleen samples of variegated squirrels (*Sciurus variegatoides*) was positive for SvarPyV1, 8/44 tested samples (6x spleen and 1x spleen and lung) from 35 Pallas’s squirrels (*Callosciurus erythraeus*) originating from Italy were positive for CeryPyV1, and 3/17 spleen samples of Prevost’s squirrels (*Callosciurus prevostii*) were positive for CprePyV1 (Table 2).

In HV tests of 118 spleen and lung samples of *Sciurus carolinensis* from the UK, Italy and USA with generic PCR, we identified 20 animals from the UK as ScarGHV1-positive (16x in spleen and 4x in spleen and lung), three Eastern grey squirrels from the USA as ScarGHV1-positive in lung and two individuals as ScarGHV2-positive, one from the UK in spleen and one from the USA in lung. ScarBHV1 was detected in lung samples of two animals from Italy. Testing 77 *Sciurus vulgaris* revealed 18 SvulBHV1-positive individuals from Germany (1x in spleen, 12x in lung and 5x in spleen and lung) and 13 from the UK (1x in spleen, 9x in lung and 3x in spleen and lung). We identified 10 out of 11 Richardson’s ground squirrels (*Urocitellus richardsonii*) to be positive for UricGHV-1 in spleen and 2/2 Eastern chipmunks (*Tamias striatus*) positive for TstrGHV1, one in spleen and one in lung. In wild *Callosciurus erythraeus* from Italy as well as *Callosciurus prevostii* from a holding in Germany, we discovered both a BHV and a GHV. Upon testing a total of 35 *Callosciurus erythraeus*, we identified 16 individuals as CeryBHV1-positive (12x in spleen, 2x in lung and 2x in spleen and lung) and two animals as CeryGHV1-positive (1x in spleen and 1x in lung). Within the 17 Prevost’s squirrels, one spleen each was detected to be positive for CpreBHV1 or CpreGHV1 (Table 3).

We did not detect any PyVs in 77 Eurasian red squirrels, 11 Richardson’s ground squirrels and two Eastern chipmunks and no HVs in seven spleen samples of variegated squirrels from German holdings. In one lung sample of an American red squirrel (*Tamiasciurus hudsonicus*) from the USA, neither PyVs nor HVs were detected (Tables 2 and 3).

Based on extended sequences that we generated for three PyVs and eight HVs (see below) we selected specific nested primers (Additional file 1) for more sensitive screening and re-tested all samples of the respective species. By this approach we observed the following prevalences: ScarPyV1 was detected in 35.2% (31/88 squirrels, 27x positive in spleen, 2x lung and 2x in spleen and lung) of *Sciurus carolinensis*, SvarPyV1 in 14.3% (1/7 spleen samples positive) of *Sciurus variegatoides*, CeryPyV1 in 42.9% (15/35 squirrels, 12x positive in spleen and 3x in spleen and lung) of *Callosciurus erythraeus* and CprePyV in 23.5% (4/17 squirrels positive in spleen) of *Callosciurus prevostii* (Table 2). For the herpesviruses, the ScarGHV1 prevalence of the *Sciurus carolinensis* from the UK was 40.8% (20/49 squirrels, 16x positive in spleen and 4x in spleen and lung) and for *Sciurus carolinensis* from the USA prevalence was 60.0% (3/5 spleen samples positive). The second gammaherpesvirus of *Sciurus carolinensis* (ScarGHV2) was detected in 2.0% (1/49 squirrels positive in spleen) and 20.0% (1/5 lung samples) of the samples from Britain and the USA, respectively. The average prevalence of ScarBHV1 amounts 5.7% (5/88 squirrels positive in spleen) and was only found in Eastern grey squirrels from Italy. The *Sciurus vulgaris* from the UK revealed a SvulBHV1-prevalence of 68.4% (13/19 squirrels, 1x positive in spleen, 9x in lung and 3x in spleen and lung) and those from Germany, 31.0% (18/58 squirrels, 1x positive in spleen, 12x in lung and 5x in spleen and lung). In *Callosciurus erythraeus* from Italy, the prevalence of CeryBHV1 was 65.7% (23/35 squirrels, 19x positive in spleen, 1x in lung and 3x in spleen and lung) and that of CeryGHV1 was 14.3% (6/35 squirrels, 5x positive in spleen and 1x in spleen and lung). Richardson’s ground squirrels from Canada showed a very high prevalence (90.9%, 10/11 spleen samples positive) of UricGHV1. The herpesvirus prevalence in Prevost’s squirrels was 5.9%: Two of seventeen samples were positive, one for CpreBHV1 and the other for CpreGHV1 (Table 3).

In this study we identified one *Sciurus variegatoides* (sample #10291) from a holding in Germany with a coinfection of VSBV-1 and SvarPyV1 and four *Callosciurus prevostii* (samples #10295, #10296, #10303 and #10304) from German holdings that were infected with VSBV-1 and CprePyV1.

### Amplification of complete polyomavirus genomes

**Fig. 1:**
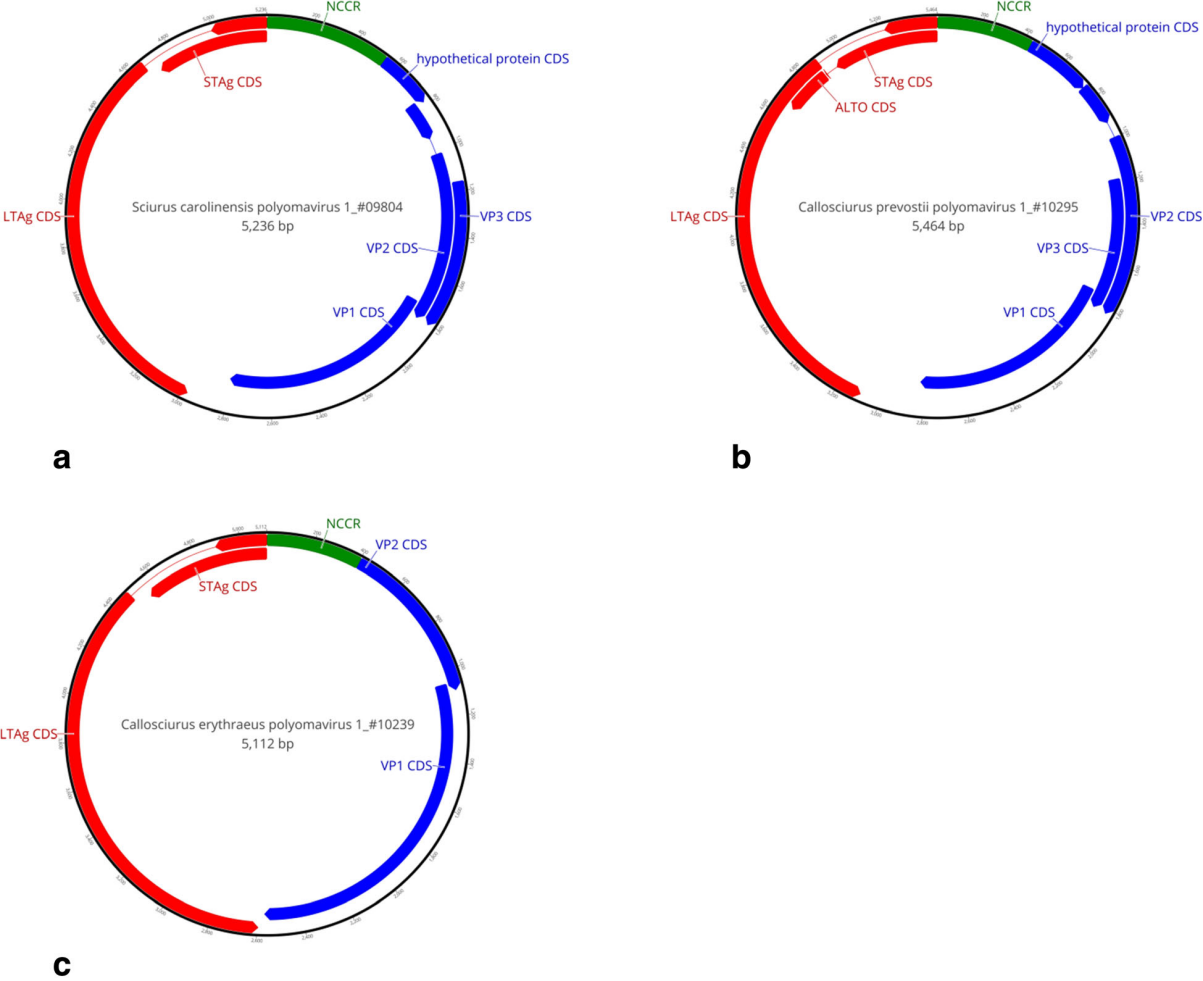
Genome organization of *Sciurus carolinensis* PyV1 (**a**), *Callosciurus prevostii* PyV1 (**b**) and *Callosciurus erythraeus* PyV1 (**c**). Putative coding regions for VP1, VP2 and VP3 are marked by blue bars; putative STAg antigen and LTAg antigen coding regions are marked by red bars, each with arrow head indicating direction of transcription. Thin lines demarcate introns. Non-coding control region (NCCR) is marked by green bars. An additional hypothetical protein CDS was predicted in the genomes of ScarPyV1 and CprePyV1

Complete genomes of ScarPyV1, CeryPyV1, and CprePyV1 were generated by nested LD-PCR using specific tail-to-tail primers (Fig. 1, Additional file 1 and Additional file 2). The LD-PCR products of around 5.2 kbp were sequenced by classical primer-walking (primers not listed) and sequences of the (i) initial generic PCR products, (ii) LD-PCR products, and (iii) specific PCR fragments mentioned above were assembled to final circular genome sequences (Fig. 1). Full PyV genomes or partial PyV sequences are listed with countries of origin and GenBank accession numbers in Table 4.

**Table 4:** Novel polyoma- and herpesvirus sequences deposited in GenBank

Virus name	Country of origin	Sample ID	Sequence length (bp)	Complete genome	GenBank accession number
CeryPyV1	Italy	#10239, #10271, #10275	5112	+	MK671087, MK671088, MK671089
CprePyV1	Germany^a^	#10295, #10296, #10304	5464	+	MK883808, MK883809, MK883810
ScarPyV1	UK	#9804, #9982, #10018	5236, 5237, 5237	+	MK671101, MK671096, MK671097
SvarPyV1	Germany^a^	#10291	213		MK671090
CeryBHV1	Italy	#10257, #10259, #10262	478		MK957142, MK957143, MK957144
CpreBHV1	Germany^a^	#10298	478		MN037512
ScarBHV1	Italy	#10197	412		MN047451
SvulBHV1	UK	#9807, #9808, #9813, #9824	3336, 3336, 3327, 3336		MK671091, MK671092, MK671093, MK671094
	Germany	#9886	3442		MK671095
CeryGHV1	Italy	#10276	472		MK957139
CpreGHV1	Germany^a^	#10305	166		not archivable in GenBank (< 200 bp)
ScarGHV1	UK	#9783, #9800, #9802	3334		MK671098, MK671099, MK671100
ScarGHV2	USA	#10179	166		not archivable in GenBank (< 200 bp)
UricGHV1	Canada	#10168, #10170, #10171, #10173, #10174, #10175	3212		MK671102, MK671103, MK671104, MK671105, MK671106, MK671107
TstrGHV1	USA	#10182, #10183	443		MK957140, MK957141

^a^animals originate from holdings

ORF analysis with Geneious 11.1.5. software revealed the typical PyV genome organization with CDS for the viral capsid proteins VP1, VP2 and VP3, the regulatory proteins STAg and LTAg (but not middle T antigen (MTag)) on the opposite strand, and the NCCR (Fig. 1). As the LTAg CDS of all other mammalian PyVs and some of the STAg CDS are interrupted by an intron, we searched for such introns in the squirrel PyV genomes in silico and experimentally, as described in detail below. In addition, the VP2 ORF of CprePyV1 and ScarPyV1 was found to be interrupted by two (CprePyV1) or three (ScarPyV1) stop codons (not shown). Therefore, splicing of the VP2-encoding late mRNA was predicted and analyzed experimentally in cell culture. In upstream direction of the VP2 coding sequence ScarPyV1 and CprePyV1 display an additional ORF in their genome. In a few PyVs of the genus *Betapolyomavirus*, e.g. SV40 and BK virus, the so-called agnoprotein is encoded at this position. Agnoprotein was identified as a regulatory protein required for efficient virus proliferation. However, the proteins putatively encoded by ScarPyV1 and CprePyV1 do not show similarities with the known agnoproteins, and their function is currently unknown.

### Identification of splice sites in early and late regions of the novel polyomaviruses

**Fig. 2:**
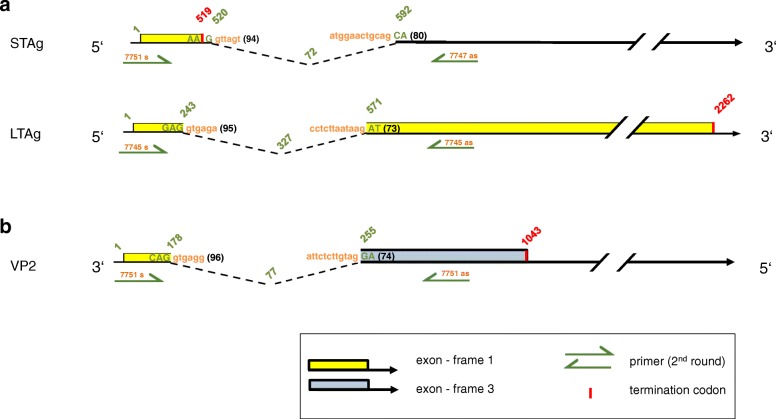
Scheme of experimentally identified spliced mRNAs of the early (**a**) and late (**b**) region of *Sciurus carolinensis* polyomavirus 1. Experimentally identified STAg, LTAg and VP2 exon sequences are depicted as colored bars, introns as dotted lines, and second-round primers (s = sense, as = antisense) for intron identification with green arrows. Exon sequences are written in green capitals and intron sequences in orange lower case. Their probability predicted by the Human Splicing Finder 3.1. is shown in brackets (score 0–100). The nucleotide positions in green font shown at intron/exon borders represent the first or last nucleotide of the exons. The nucleotide positions in red font represent the last nucleotide of the termination codon

Splice donor and acceptor sites with high Human Splicing Finder (HSF) rating (75–95) and conserved in sequence and position compared to related PyVs were identified in LTAg CDS of all three squirrel PyVs. In addition, splice sites were predicted for STAg mRNA of CprePyV1, with the splice donor site interrupting the stop codon. The introns of STAg mRNA of ScarPyV1 and CeryPyV1 were found to be located after the stop codon. Finally, an intron was predicted for the VP2 CDS of CprePyV1 and ScarPyV1 explaining the occurrence of an interrupted CDS (Fig. 2 and Table 5).

**Table 5:** Splice donor and acceptor sites in the early and late regions of the novel polyomaviruses

Polyomavirus	CDS^a^	Splice donor site EXON|intron^b^	Splice acceptor site intron|EXON^b^
Sciurus carolinensis polyomavirus 1	STAg	AAG|gttagt (94)	atggaactgcag|CA (80)
	LTAg	GAG|gtgaga (95)	cctcttaataag|AT (73)
	VP2	CAG|gtgagg (96)	attctcttgtag|GA (85)
Callosciurus prevostii polyomavirus 1	STAg	ATG|gtgagt (93)	tacctttaacag|AT (85)
	LTAg	GAG|gtaaaa (83)	tacctttaacag|AT (85)
	VP2	GAG|gtaaga (96)	ttccttttgtag|GA (87)
Callosciurus erythraeus polyomavirus 1	LTAg	GAG|gtacgt (89)	tgcttctttcag|GA (93)

^a^*CDS* coding sequence

^b^score (0–100; in brackets) of similarity to the splice consensus site generated by the Human Splicing Finder 3.1

Experimental confirmation of the splice sites predicted for ScarPyV1 was performed with an approach that was used previously for splice site analysis of two human PyVs [54]. First we transfected either the early or late region with flanking sequences of ScarPyV1 (from sample #9804) into monkey Vero cells and murine NIH/3T3 cells, isolated mRNA at the day of transfection and at days 1, 2.5 and 6 after transfection, and synthesized cDNA. Thereafter, a nested PCR was performed with primers that flank the putative introns (Additional file 1). By sequencing of the PCR products, the splice sites predicted for both the early (STAg and LTAg CDS) and the late region (VP2 CDS) were confirmed in Vero and NIH/3T3 cells as described in detail below.

With two nested PCRs that respectively span 300 bp and 500 bp in the early region around the predicted STAg intron, a spliced mRNA was detected in Vero and NIH/3T3 cells that displays an unspliced CDS of 519 bp and encodes the predicted STAg of 172 amino acids (aa). Behind the stop codon (nucleotide (nt) 517–519), a short intron of 72 nt was observed (Fig. 2a and Table 5). With PCR that spans 800 bp in the early region around the predicted LTAg intron, a spliced mRNA was detected in Vero and NIH/3T3 cells from which a spliced CDS of 1.935 kbp (exon 1 and 2 in frame + 1) was inferred. It encodes an LTAg of 644 aa (Table 5 and Fig. 2a). The ScarPyV1-encoded TAgs share the 81 N-terminal aa.

Finally, we performed a PCR that spans 400 bp in the late region around the predicted VP2 intron. A spliced CDS was amplified from cDNA that displays two exons (Table 5 and Fig. 2b).

The experimental results generated for ScarPyV1 were completely in line with the theoretical predictions, i.e., all three predicted introns were detected in both tested cell lines and at different time points of harvesting. The splice sites predicted for LTAg, STAg, and VP2 CDS of CprePyV1 (Table 5) were not studied experimentally because the CprePyV1 genome is closely related to that of ScarPyV1 (see phylogenetic tree analysis below) and the splice sites of both PyVs are conserved in position and sequence. Likewise, splice sites predicted for CeryPyV1 were not studied experimentally as they are conserved in position and sequence with those of the closely related *Philantomba monticola* PyV1, whose sites were experimentally confirmed earlier [65].

### Determination of glycoprotein B and extended DNA polymerase nucleotide sequences of herpesviruses

To extend the DPOL gene sequence information for each discovered HV in upstream direction into the adjacent gB CDS, all samples HV-positive in the DPOL PCR were re-evaluated with generic PCRs that were reported earlier [44, 51, 52, 66] to target the gB sequences of either BHVs or GHVs (Additional file 1 and Additional file 3). These generic gB PCRs were less sensitive compared to the generic DPOL PCR, as we amplified a partial gB sequence of ScarGHV1 from only one lung sample of 20 spleen and seven lung samples that had been positive in DPOL PCR. SvulBHV1 gB sequence was amplified from two spleen and three lung samples of 39 DPOL PCR-positive samples and UricGHV1 gB sequence was amplified from all 10 DPOL PCR-positive spleen samples. Next, we closed the sequence gap between the gB and the DPOL sequence (approximately 3.2 kbp) for each of the three HVs with LD-PCR and sequenced the gB-to-DPOL product by classical primer walking. This led to five continuous SvulBHV1 sequences with a length of 3.327–3.442 kbp, three ScarGHV1 sequences of 3.334 kbp, and five UricGHV1 sequences of 3.212 kbp (Table 4).

For ScarBHV1, ScarGHV2, TstrGHV1, CpreBHV1, CpreGHV1, CeryBHV1 and CeryGHV1 the generic gB PCR did not produce the desired gB fragment. Therefore we extended the short DPOL sequences from around 170 bp to > 400 bp by using the outer sense primer of the generic DPOL PCR (285-S DFA; Additional file 1) in combination with two virus-specific antisense primers (Additional file 1) in hemi-nested PCR format. This approach was successful for ScarBHV1 (412 bp; from one sample), CeryBHV1 (478 bp; from three samples), CeryGHV1 (472 bp from one sample), CpreBHV1 (478 bp; from one sample) and TstrGHV-1 (443 bp from two samples), and failed for CpreGHV1 and ScarGHV2. The partial HV sequences are listed with countries of origin and GenBank accession numbers in Table 4.

### Phylogenetic analysis of conserved amino acid blocks of the polyomavirus LTAg and VP1 sequences

**Fig. 3:**
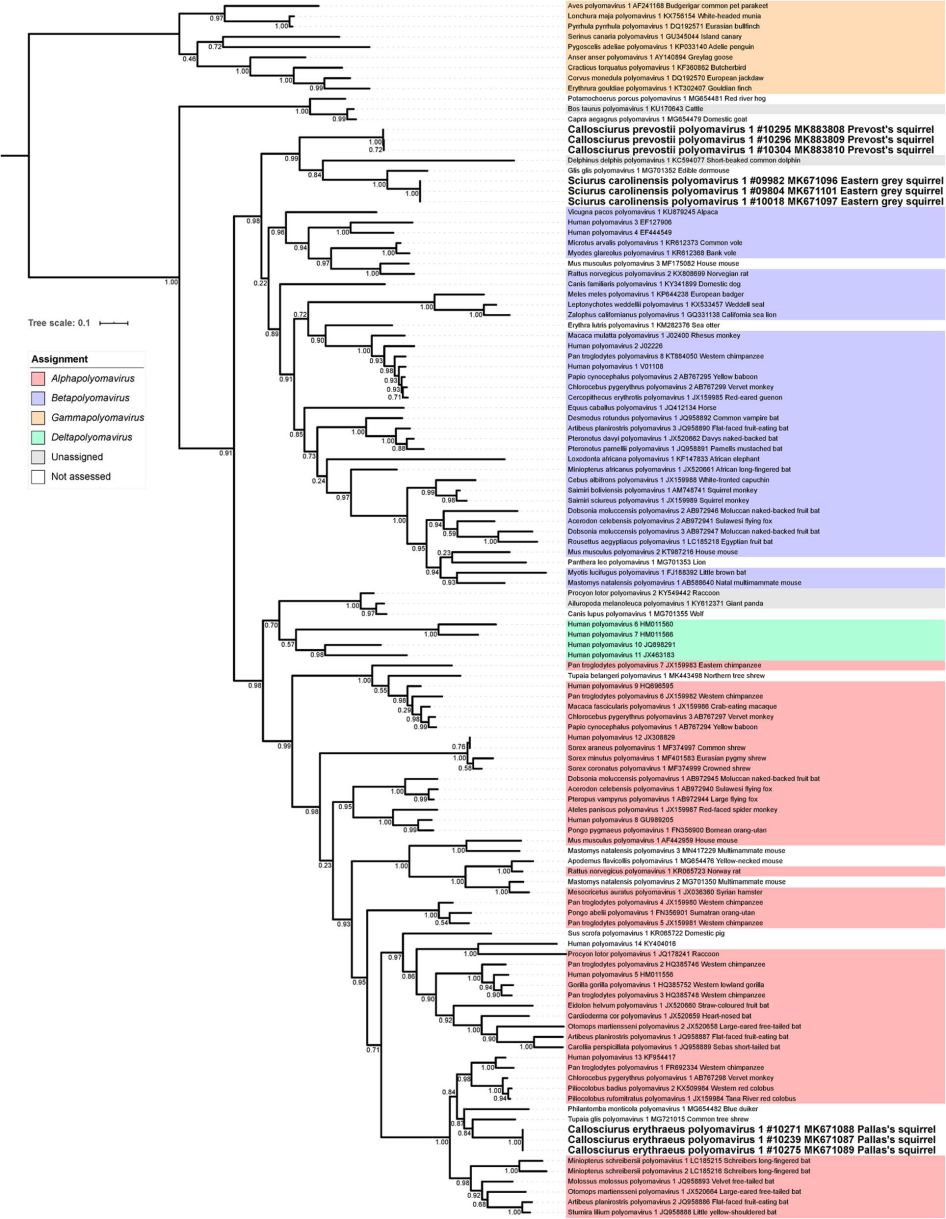
Maximum-likelihood (ML) tree analysis of polyomaviruses based on conserved amino acid blocks of the polyomavirus LTAg sequences. Phylogenetic relationships of polyomaviruses, including classification of the novel viruses, based on conserved amino acid blocks of LTAg sequence. Polyomaviruses are denoted by Latin taxonomic names of their hosts, GenBank accession number, common name of the species and – in case of the new viruses – sample ID. For International Committee on Taxonomy of Viruses (ICTV)-recognized species, virus genera are indicated by colors. Viruses newly identified in this study are given in bold font. Branch support was assessed using Shimodaira-Hasagawa-like approximate likelihood ratio tests (SH-like aLRT)

For evolutionary conclusions and taxonomical classification phylogenetic analyses were done. The ML and MCC trees based on PyV LTAg aa sequences (Fig. 3 and Additional file 4) are quite similar and allow the following conclusions: the novel PyVs can be tentatively assigned to different genera within the family *Polyomaviridae*, as ScarPyV1 and CprePyV1 nest within the diversity of genus *Betapolyomavirus* and CeryPyV1 in the genus *Alphapolyomavirus*. Within the betapolyomaviruses the three ScarPyV1 (GenBank Accession numbers MK671096, MK671097 and MK671101) and the three CprePyV1 (GenBank Accession numbers MK883808 - MK883810) cluster together in a well-supported monophyletic group, which also comprises another rodent polyomavirus, Glis glis polyomavirus 1 (GgliPyV1, GenBank Accession number MG701352), and *Delphinus delphis* polyomavirus 1 (DdelPyV1, GenBank Accession number KC594077). The close evolutionary relationship of these viruses is strengthened by the observation that their VP2 CDS is interrupted by an intron. This splicing event within the VP2 CDS has been either experimentally verified (*Sciurus carolinensis* polyomavirus 1 (this study) and Glis glis polyomavirus 1 [65]) or predicted in silico for *Delphinus delphis* polyomavirus 1*,* and *Callosciurus prevostii* polyomavirus 1 as the VP2 CDS of all clade members comprise highly conserved splice donor and acceptor motifs with high score (> 75) in the Human Splicing Finder 3.1.

All three representatives of the novel CeryPyV1 cluster together within the genus *Alphapolyomavirus*. Although their exact phylogenetic placement is uncertain, they belong to a well-supported clade of polyomaviruses infecting hosts of the orders Artiodactyla, Chiroptera, Primates and Scandentia (Fig. 3 and Additional file 4).

As expected and reported many times, the VP1-based ML and MCC trees (Additional file 5 and Additional file 6) support clades that differ from those delineating genera in the LTAg-based analyses [41]. In these trees, ScarPyV1 and CprePyV1 also formed a weakly supported monophyletic group with GgliPyV1 and DdelPyV1 but this group also included *Canis familiaris* polyomavirus 1 (GenBank Accession number KY341899). This virus may also have a spliced VP2 as it shares conserved VP2 splice sites with the other four polyomaviruses. The evolutionary position of SvarPyV1 (from the variegated squirrel) had to be allocated in the phylogenetic trees based on VP1 (Additional file 5 and Additional file 6), because only a partial VP1 sequence was identified. The single SvarPyV1 sequence clusters within the genus *Alphapolyomavirus* and is a sister virus to a PyV group consisting of an organ-utan and two chimpanzee PyVs. This phylogenetic allocation and the tentative host association of SvarPyV1 will need confirmation, once additional SvarPyV1 sequences from several animals and a complete SvarPyV1 LTAg sequence are available.

### Phylogenetic analysis of conserved amino acid blocks of herpesvirus partial DPOL and gB sequences

**Fig. 4:**
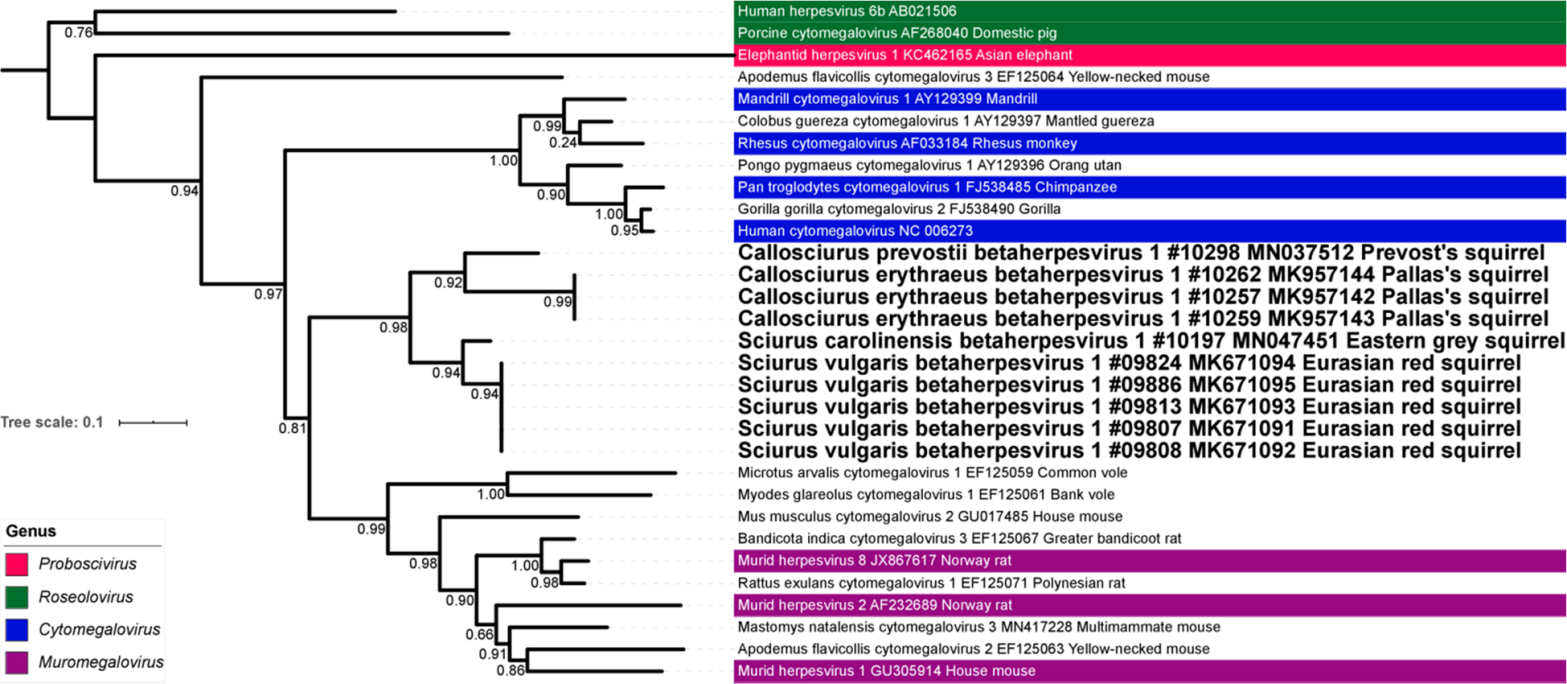
Maximum-likelihood (ML) tree analysis of betaherpesviruses based on conserved amino acid blocks of the DPOL sequences. Phylogenetic relationships of betaherpesviruses, including classification of the novel viruses, based on conserved amino acid blocks of DPOL sequence. Betaherpesviruses are denoted by Latin taxonomic names or common names of their hosts, GenBank accession number, common name of the species and – in case of the new viruses – sample ID. For ICTV-recognized species, virus genera are indicated by colors. Viruses newly identified in this study are given in bold font. Branch support was assessed using Shimodaira-Hasagawa-like approximate likelihood ratio tests (SH-like aLRT)

The ML and MCC trees based on partial DPOL sequences of BHVs (Fig. 4 and Additional file 7) are very similar and show that the four identified squirrel BHVs form a separate cluster that is associated with moderate support to a clade comprising rodent HVs of the genus *Muromegalovirus* and other currently unclassified rodent HVs. The ML and MCC trees based on partial gB (Additional file 8 and Additional file 9) are very similar to the DPOL-based trees but only the *Sciurus vulgaris* betaherpesvirus 1 sequences are included as for the other three BHVs gB sequences were not available.

**Fig. 5:**
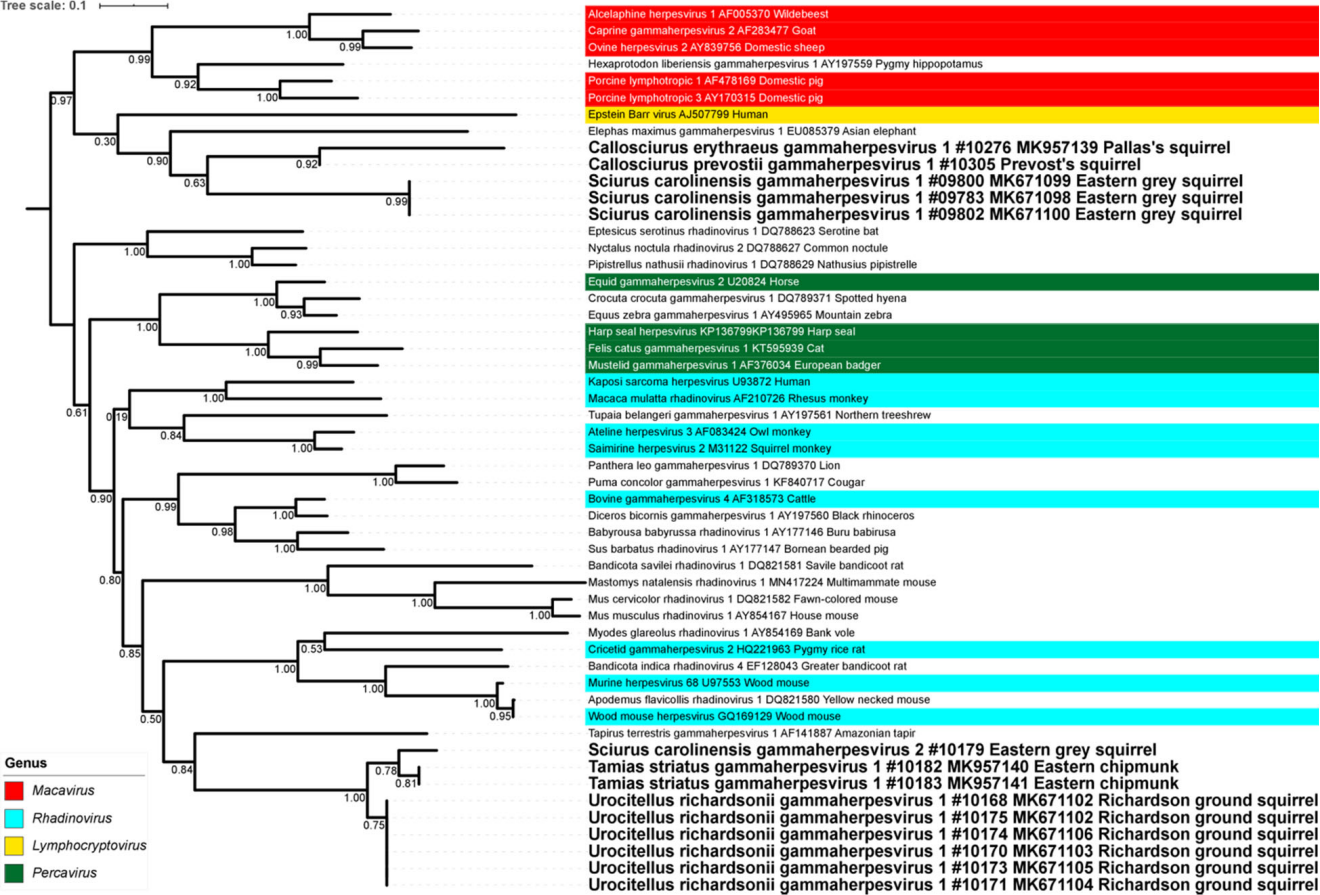
Maximum-likelihood (ML) tree analysis of gammaherpesviruses based on conserved amino acid blocks of the DPOL sequences. Phylogenetic relationships of gammaherpesviruses, including classification of the novel viruses, based on conserved amino acid blocks of DPOL sequence. Gammaherpesviruses are denoted by Latin taxonomic names or common names of their hosts, GenBank accession number, common name of the species and – in case of the new viruses – sample ID. For ICTV-recognized species, virus genera are indicated by colors. Viruses newly identified in this study are given in bold font. Branch support was assessed using Shimodaira-Hasagawa-like approximate likelihood ratio tests (SH-like aLRT)

The ML and MCC phylogenetic trees based on partial DPOL (Fig. 5 and Additional file 10) and partial gB of GHVs (Additional file 11 and Additional file 12) are also quite similar and show that the squirrel GHVs form two separate groups. One group nests within GHVs of the genus *Rhadinovirus* and forms a weakly supported clade with a group of rodent HVs (in all trees) and a rhadinovirus of the South American tapir (only in DPOL-based trees). The other squirrel HV group nests within a clade that comprises ungulate GHVs of the genus *Macavirus*, a GHV of African elephant and the human Epstein-Barr virus (species *Human herpes virus 4*).

## Discussion

The screening of more than 200 squirrels from holdings and sampled wildlife, either indigenous or introduced species, from five countries on two continents resulted in the discovery of four novel PyVs, four novel BHVs and six novel GHVs. Phylogenetic analysis allocated the novel squirrel PyVs to the genera *Alphapolyomavirus* and *Betapolyomavirus*, the novel BHVs to the herpesvirus genus *Muromegalovirus*, and the GHVs to the genera *Rhadinovirus* and *Macavirus*. This first description of PyVs, BHVs and GHVs in squirrels of different species increased our knowledge (e.g. [51, 65] on the diversity of PyVs and HVs in rodents. In particular, some of these viruses represent novel highly divergent lineages or sublineages in the corresponding phylogenetic trees.

The results of our PyV and HV investigations furthermore show that (i) the virus-specific nested PCRs have a higher sensitivity than the generic PCRs, thereby increasing the number of PyV- and HV-positive samples and positive individuals and (ii) virus detection was more frequent in spleen, compared to lung samples. The adapted methods were used here for a first prevalence estimation for some of these viruses, allowing an initial comparison of the prevalences in indigenous and introduced populations.

Based on the prevalence of the different viruses and their detection in exclusively only one respective squirrel species, it is likely that the viruses are all species-specific and were detected in their respective natural hosts. The fact that the novel squirrel PyVs and HVs or closely related ones were not detected previously in other host species may further strengthen this assumption of host specificity. These investigations thereby improve our knowledge on the host specificity of PyVs and HVs [29, 41].

Our study indicated a broad geographical distribution of some of the novel viruses: ScarPyV1 was detected in the Eastern Grey squirrel from the original range in North America, but also in introduced populations in Great Britain and Italy. Similarly, both ScarGHV1 and ScarGHV2 were detected in the original North American and the introduced British populations. The detection of SvulBHV1 in Eurasian red squirrels from Germany and Scotland might be explained by the long, interlinked history of Eurasian red squirrels on the British Isles with other European populations as discussed previously for the detection of Eurasian red squirrel-associated Squirrel adenovirus 1 (SqAdV-1) strains of high sequence similarity in Germany and Scotland [13].

For three PyVs complete genomes were generated and splice sites were experimentally determined for one of these novel viruses. As reported earlier [65], such experimental determination is critically important for annotation of coding PyV sequences. The search for the reservoir of VSBV-1 within this study resulted in solely negative findings, although additional squirrel species were investigated. This confirms results of our previous study that the Eastern grey squirrel is most likely not the reservoir of bornaviruses [21] and suggests that the Pallas’s squirrel (*Callosciurus erythraeus*) is also not a reservoir host for known bornaviruses, at least in the investigated introduced Italian population. It is still unclear and requires further investigation why squirrels of two different subfamilies in German private and zoo holdings which were imported from different geographic origins (*Sciurus variegatoides* and *Callosciurus prevostii*) harbor VSBV-1 sequences [21] of such high similarity. Further screening approaches, including squirrels and other small mammals, will focus on the identification of other possible reservoir hosts of orthobornaviruses in the future. Thereby, it should be evaluated if (i) VSBV-1 was imported with squirrels from Central America or South East Asia and afterwards spread in the German squirrel holdings and holdings in The Netherlands and Croatia, or if (ii) another, yet unknown reservoir host of VSBV-1 exists in Central Europe. Furthermore, experimental infection studies will be done to evaluate the VSBV-1 susceptibility of different squirrel species.

In this study we identified SvarPyV1 in a *Sciurus variegatoides* from a German holding that was tested positive for VSBV-1 in a previous study and CprePyV1 in four *Callosciurus prevostii* from German holdings that were also tested VSBV-1-positive before [17, 21, 22]. These observations indicate viral coinfections in squirrels, confirming results from previous studies in other rodents [67–69]. Evidence for bi-directional interplay of viral and/or bacterial agents, e.g. altering the host’s susceptibility, the disease progression, severity of the disease and the host’s immune response in rodents have been reported [70, 71]. However, it is currently unknown if the agents investigated in the current study affect each other, and we have no direct evidence if the detected agents are causing clinical signs or pathologic alterations in the investigated squirrels.

## Conclusions

This is the first report on molecular identification and sequence characterization of PyVs and HVs in rodents of the family Sciuridae. These findings will allow further targeted screenings of squirrels of the investigated species to analyze the role that these novel viruses play on the population dynamics and competitive interactions in wildlife squirrel populations. Furthermore, the origin of these novel viruses and their spatially and temporally driven evolution in indigenous and introduced populations of grey squirrels, Pallas’s squirrels and Prevost’s squirrels and questions regarding the interactions of different agents in squirrels are interesting topics for future investigations.

## Supplementary information

**Additional file 1.** PCR assays for detection of borna-, polyoma- and herpesviruses and mycoplasma.

**Additional file 2.** Flow chart of multi-level PCR analysis for detection of squirrel polyomaviruses. Generic nested VP1 PCR (second-round product displayed as magenta-coloured bar) with degenerate primers was conducted. For full genome amplification, this was followed by specific nested long-distance PCR (LD-PCR; second-round product of approximately 5 kbp shown as red bar) and overlapping standard nested PCR (second-round product of approximately 800 bp shown as green bar) with specific primers. Grey bars represent coding sequences, black bar the non-coding control region.

**Additional file 3.** Flow chart of multi-level PCR analysis for detection of squirrel herpesviruses. Generic nested DPOL PCR (product: bar in magenta) with degenerate primers was carried out. For extended sequence determination, this was followed by generic gB PCR (blue) with degenerate primers and subsequent long-distance PCR (LD-PCR) (red) with specific primers. Products of the second PCR rounds are shown. The sequences of the generic DPOL PCR product and the extended DPOL PCR product build a contiguous sequence of 0.4–0.5 kbp (black). The sequences of the generic gB and the generic DPOL PCR product build together with the LD-PCR-derived sequence a contiguous sequence of approximately 3.3 kbp (black). On top of the figure, coding sequences are displayed by grey bars. The arrow heads indicate the direction of transcription.

**Additional file 4.** Maximum clade credibility tree analysis of polyomaviruses based on conserved amino acid blocks of the LTAg sequences. Phylogenetic relationships of polyomaviruses, including classification of the novel viruses, based on conserved amino acid blocks of LTAg sequence. Branch support values displayed at the nodes correspond to their posterior probability. For further details see legend of Fig. 3.

**Additional file 5.** Maximum likelihood tree analysis of polyomaviruses based on conserved amino acid blocks of the VP1 sequences. Phylogenetic relationships of polyomaviruses, including classification of the novel viruses, based on conserved amino acid blocks of VP1 sequence. Branch support values displayed at the nodes were assessed using Shimodaira-Hasagawa-like approximate likelihood ratio tests (SH-like aLRT). For further details see legend of Fig. 3.

**Additional file 6.** Maximum clade credibility tree analysis of polyomaviruses based on conserved amino acid blocks of the VP1 sequences. Phylogenetic relationships of polyomaviruses, including classification of the novel viruses, based on conserved amino acid blocks of VP1 sequence. Branch support values displayed at the nodes correspond to their posterior probability. For further details see legend of Fig. 3.

**Additional file 7.** Maximum clade credibility tree analysis of betaherpesviruses based on conserved amino acid blocks of the DPOL sequences. Phylogenetic relationships of betaherpesviruses, including classification of the novel viruses, based on conserved amino acid blocks of DPOL sequence. Branch support values displayed at the nodes correspond to their posterior probability. For further explanation see legend of Fig. 4.

**Additional file 8.** Maximum likelihood tree analysis of betaherpesviruses based on conserved amino acid blocks of the gB sequences. Phylogenetic relationships of betaherpesviruses, including classification of the novel viruses, based on conserved amino acid blocks of gB sequence. Branch support values displayed at the nodes were assessed using Shimodaira-Hasagawa-like approximate likelihood ratio tests (SH-like aLRT). For further explanation see legend of Fig. 4.

**Additional file 9.** Maximum clade credibility tree analysis of betaherpesviruses based on conserved amino acid blocks of the gB sequences. Phylogenetic relationships of betaherpesviruses, including classification of the novel viruses, based on conserved amino acid blocks of gB sequence. Branch support values displayed at the nodes correspond to their posterior probability. For further explanation see legend of Fig. 4.

**Additional file 10.** Maximum clade credibility tree analysis of gammaherpesviruses based on conserved amino acid blocks of the DPOL sequences. Phylogenetic relationships of gammaherpesviruses, including classification of the novel viruses, based on conserved amino acid blocks of DPOL sequence. Branch support values displayed at the nodes correspond to their posterior probability. For further explanation see legend of Fig. 5.

**Additional file 11.** Maximum likelihood tree analysis of gammaherpesviruses based on conserved amino acid blocks of the gB sequences. Phylogenetic relationships of gammaherpesviruses, including classification of the novel viruses, based on conserved amino acid blocks of gB sequence. Branch support values displayed at the nodes were assessed using Shimodaira-Hasagawa-like approximate likelihood ratio tests (SH-like aLRT). For further explanation see legend of Fig. 5.

**Additional file 12.** Maximum clade credibility tree analysis of gammaherpesviruses based on conserved amino acid blocks of the gB sequences. Phylogenetic relationships of gammaherpesviruses, including classification of the novel viruses, based on conserved amino acid blocks of gB sequence. Branch support values displayed at the nodes correspond to their posterior probability. For further explanation see legend of Fig. 5.

## Data Availability

All data generated or analyzed during this study are included in this published article. Sequences of the novel viruses have been deposited in GenBank with accession numbers MK671087, MK671088, MK671089, MK671090, MK671091, MK671092, MK671093, MK671094, MK671095, MK671096, MK671097, MK671098, MK671099, MK671100, MK671101, MK671102, MK671103, MK671104, MK671105, MK671106, MK671107, MK883808, MK883809, MK883810, MK957139, MK957140, MK957141, MK957142, MK957143, MK957144, MN037512, MN047451.

## Abbreviations

aa: Amino acid
as: Antisense
BHVs: Betaherpesviruses
BLASTn: Nucleotide Basic Local Alignment Search Tool
BMCMC: Bayesian Markov chain Monte Carlo
BoDV-1: Borna Disease Virus 1
BoDV-2: Borna Disease Virus 2
bp: Base pairs
CDS: Coding sequence
CeryBHV1: *Callosciurus erythraeus* betaherpesvirus 1
CeryGHV1: *Callosciurus erythraeus* gammaherpesvirus 1
CeryPyV1: *Callosciurus erythraeus* polyomavirus 1
CpreBHV1: *Callosciurus prevostii* betaherpesvirus 1
CpreGHV1: *Callosciurus prevostii* gammaherpesvirus 1
CprePyV1: *Callosciurus prevostii* polyomavirus 1
DdelPyV1: *Delphinus delphis* polyomavirus 1
DMEM: Dulbecco’s minimal Eagle medium
DMSO: Dimethyl sulfoxide
dNTP: Dideoxynucleoside triphosphate
DPOL: DNA polymerase
ECACC: European Collection of Authenticated Cell Cultures
FCS: Fetal calf serum
gB: Glycoprotein B
GgliPyV1: Glis glis polyomavirus 1
GHVs: Gammaherpesviruses
HSF: Human Splicing Finder
HVs: Herpesviruses
ICTV: International Committee on Taxonomy of Viruses
kb: Kilobases
kbp: Kilobase pairs
LD-PCR: Long-distance PCR
LTAg: Large T-antigen
MCC: Maximum clade credibility
MCPyV: Merkel cell polyomavirus
ML: Maximum likelihood
MTAg: Middle T-antigen
NCCR: Non-coding control region
nt: Nucleotide
ORF: Open reading frame
PCR: Polymerase chain reaction
PhyML-SMS: PhyML v3 with smart model selection
PST: Posterior set of trees
PyVs: Polyomaviruses
RT-qPCR: Reverse transcription-quantitative polymerase chain reaction
s: Sense
ScarBHV1: *Sciurus carolinensis* betaherpesvirus 1
ScarGHV1: *Sciurus carolinensis* gammaherpesvirus 1
ScarGHV2: *Sciurus carolinensis* gammaherpesvirus 2
ScarPyV1: *Sciurus carolinensis* polyomavirus 1
SH-like aLRT: Shimodaira-Hasegawa-like approximate likelihood ratio test
SqAdV-1: Squirrel adenovirus 1
STAg: Small T-antigen
SvarPyV1: *Sciurus variegatoides* polyomavirus 1
SvulBHV1: *Sciurus vulgaris* betaherpesvirus 1
TstrGHV1: *Tamias striatus* gammaherpesvirus 1
UricGHV1: *Urocitellus richardsonii* gammaherpesvirus 1
VSBV-1: Variegated squirrel bornavirus 1
