# Supplementary material for: Search for polyoma-, herpes-, and bornaviruses in squirrels of the family Sciuridae
Source: Virol J. 2020 Mar 27;17:42. doi: 10.1186/s12985-020-01310-4 (PMC7099801; doi:10.1186/s12985-020-01310-4)
Supplement: Supplementary file 1 — Additional file 1. PCR assays for detection of borna-, polyoma- and herpesviruses and mycoplasma. [file 12985_2020_1310_MOESM1_ESM.docx]

**Additional file 1. PCR assays for detection of borna-, polyoma- and herpesviruses and mycoplasma**

| **PCR-Assay** | **Primer name** | **PCR round** | **target gene** | | **Sequence (5‘ to 3‘)** | | | | **Fragment size (bp)** | **Annealing temperature (°C)** | | **Reference** | |
| --- | --- | --- | --- | --- | --- | --- | --- | --- | --- | --- | --- | --- | --- |
| **Bornavirus** |  |  |  | |  | | | |  |  | |  | |
| Squirrel Borna Virus-1 mix 10 (RT-qPCR) | BDV-like-7685-F | - | X/P | | ACTCTCTTCGTCCTCCAATG | | | | 64 | 57 | | (17) | |
|  | BDV-like-7448-R |  |  |  | CATCAGACGGCTCAATGGCA | | | |  |  |  |  |  |
|  | BDV-like-7712-FAM |  |  |  | FAM-AGACTCGAGGGGCGCGATGCCAT-BHQ1 | | | |  |  |  |  |  |
| broad-range orthobornavirus mix 7.2 (RT-qPCR) | Borna-1319-F | - | X/P | | CGCGACCMTCGAGYCTRGT | | | | 211 | 57 | | (28) | |
|  | Borna-1529-R |  |  |  | GACARCTGYTCCCTTCCKGT | | | |  |  |  |  |  |
|  | Borna-1471.2-FAM |  |  |  | FAM-AAGAAYCCHTCCATGATCTCMGAYCMAGA-BHQ1 | | | |  |  |  |  |  |
| **Polyomavirus** |  |  |  | |  | | | |  |  | |  | |
| generic polyomavirus VP1 PCR | 4956s | 1 | PyV VP1 CDS | | CCTGATCCTTCTA(R/I^a^)(R/I)AATGA(R/I)AA | | | | 800-1100 | 46 | | (48) | |
|  | 4956as |  |  |  | AATAAGAAGCATCAGAT(N/I^b^)TTYCC(N/I)CC | | | |  |  |  |  |  |
|  | 4957s | 2 |  |  | ATGAAAATGGGGTTGGCCC(N/I)CT(N/I)TGYAARG | | | | 250-270 | 46 | |  |  |
|  | 4957as |  |  |  | CCCTCATAAACCCGAACYTCYTC(H/I)ACYTG | | | |  |  |  |  |  |
| ScarPyV1 specific nested VP1 PCR | 7674s | 1 | ScarPyV1b VP1 CDS | | AGGGAAAATCATGCCTCACA | | | | 879 | 59 | | this study | |
|  | 7674as |  |  |  | GGCCTGGAAAGAGACCCTTG | | | |  |  |  |  |  |
|  | 7675s | 2 |  |  | ACAAGGCTGTTTTGGATAGGGA | | | | 822 | 60 | |  |  |
|  | 7675as |  |  |  | AAAAACATGTGTCGCCCACC | | | |  |  |  |  |  |
| ScarPyV1a LD-PCR^c^ | 7761s | 1 | ScarPyV1a genome | | | TGTACTCCAAGGGGAAGGGT | | | approx. 5000 | 55 | | | this study |
|  | 7761as |  |  |  |  | ACACAAGGGACCAACTCCAT | | |  |  |  |  |  |
|  | 7762s | 2 |  |  |  | CCACTACCTCAGACGTTGGC | | | approx. 5000 | 55 | | |  |
|  | 7762as |  |  |  |  | TTGGAGAACAGGTGGGGTCT | | |  |  |  |  |  |
|  | 7763s | 3 |  |  |  | TGCCCTTCTCCTTTATTGCTGA | | | approx. 5000 | 55 | | |  |
|  | 7763as |  |  |  |  | TGCACGCCTCCATGTAAAGT | | |  |  |  |  |  |
|  |  |  |  | | |  | | |  |  | | |  |
|  |  |  |  | | |  | | |  |  | | |  |
| ScarPyV1b LD-PCR | 7663s | 1 | ScarPyV1b genome | | | GGAAGCGCCTGGTGAAGAA | | | approx. 5000 | 55 | | | this study |
|  | 7663as |  |  |  |  | GAGCCCTCTCCAATAAGCCC | | |  |  |  |  |  |
|  | 7664s | 2 |  |  |  | CCTGGTGAAGAATCCCTATCCA | | | approx. 5000 | 55 | | |  |
|  | 7664as |  |  |  |  | AAGCCCTATTGCCCTGATCT | | |  |  |  |  |  |
|  | 7665s | 3 |  |  |  | ATCCCTATCCAATTAATTCCCTTCT | | | approx. 5000 | 55 | | |  |
|  | 7665as |  |  |  |  | CCCTGATCTACTTGCCAGCC | | |  |  |  |  |  |
| ScarPyV1 STAg intron-spanning PCR | 7748s | 1 | STAg intron plus flanking sequences | | | TGCCATCAAATCAGGTGCAA | | | 355 | 58 | | | this study |
|  | 7744as |  |  |  |  | TTGCACTGGTTCCATCCTCC | | | 578 |  |  |  |  |
|  | 7749s | 2 |  |  |  | AGTTTAGTGTGGTTGAGATGCT | | | 319 | 60 | | |  |
|  | 7745as |  |  |  |  | TTCCATCCTCCTCCTCGGG | | | 560 |  |  |  |  |
| ScarPyV1 LTAg intron-spanning PCR | 7744s | 1 | LTAg intron plus flanking sequences | | | AGGGGGAGATGGAGAAAAGA | | | 578 | 59 | | | this study |
|  | 7744as |  |  |  |  | TTGCACTGGTTCCATCCTCC | | |  |  |  |  |  |
|  | 7745s | 2 |  |  |  | TGGAGAAAAGATGAAGAGAATGAATGT | | | 560 | 61 | | |  |
|  | 7745as |  |  |  |  | TTCCATCCTCCTCCTCGGG | | |  |  |  |  |  |
| ScarPyV1 VP2 intron-spanning PCR | 7750s | 1 | VP2 intron plus flanking sequences | | | TGGGAGCTGTTATTTCATTGATTCT | | | 397 | 60 | | | this study |
|  | 7750as |  |  |  |  | GGTGGACACAGGTATAGCGT | | |  |  |  |  |  |
|  | 7751s | 2 |  |  |  | TGATGCTGTTGAGATAGCTGCT | | | 332 | 59 | | |  |
|  | 7751as |  |  |  |  | AATCCAACAGCCACGAAAGG | | |  |  |  |  |  |
| CeryPyV1 LD-PCR | 7758s | 1 | CeryPyV1 genome | | | CCCTATCCCATTACTTCTTTGTTGG | | | approx. 5000 | 55 | | | this study |
|  | 7758as |  |  |  |  | ACATATAATCCACAAATATCTGCAGAA | | |  |  |  |  |  |
|  | 7759s | 2 |  |  |  | CCCATTACTTCTTTGTTGGGAAGTT | | | approx. 5000 | 55 | | |  |
|  | 7759as |  |  |  |  | CCACAAATATCTGCAGAAGACACA | | |  |  |  |  |  |
|  | 7760s | 3 |  |  |  | TGGGAAGTTTGTTTTCAGGACT | | | approx. 5000 | 55 | | |  |
|  | 7760as |  |  |  |  | TGCAGAAGACACAAAAAGTCCA | | |  |  |  |  |  |
| CeryPyV1 specific nested VP1 PCR | 7767s | 1 | CeryPyV1 VP1 CDS | | | TTTTTGCAGTGGGAGGGGAA | | 620 | | 60 | | this study | |
|  | 7767as |  |  |  |  | GGTCTGGATCTCCTGGTAACC | |  |  |  |  |  |  |
|  | 7768s | 2 |  |  |  | TTGCAGTGGGAGGGGAAC | | 594 | | 60 | |  |  |
|  | 7768as |  |  |  |  | TCCATTCCCTGATAGACTCTAACT | |  |  |  |  |  |  |
|  |  |  |  | | |  | |  | |  | |  | |
| CprePyV1 LD-PCR | 7799-s | 1 | CprePyV1 genome | | | CCTTATCCAGTAAGCACACTGC | | approx. 5000 | | 55 | | this study | |
|  | 7799-as |  |  |  |  | AGGCCTCTCCAGTATGCTCT | |  |  |  |  |  |  |
|  | 7800-s | 2 |  |  |  | GCACACTGCTAACTTCCTTGT | | approx. 5000 | | 55 | |  |  |
|  | 7800-as |  |  |  |  | CTGAGTCTATCTGCATCCCACA | |  |  |  |  |  |  |
|  | 7801-s | 3 |  |  |  | TGCTAACTTCCTTGTTTAATGGGC | | approx. 5000 | | 55 | |  |  |
|  | 7801-as |  |  |  |  | GCATCCCACAAATGTCTGCA | |  |  |  |  |  |  |
| CprePyV1 specific nested VP1 PCR | 7822-s | 1 | CprePyV1 CDS | | | CGTGCCCTCTCTTATGAATCA | | 856 | | 60,5 | | this study | |
|  | 7822-as |  |  |  |  | GGACTACCCCGCTTGGCC | |  |  |  |  |  |  |
|  | 7823-s | 2 |  |  |  | TGCTAATGGCCCAGGTATTCC | | 800 | | 61 | |  |  |
|  | 7823-as |  |  |  |  | CTTGGCCGCAACGTGAGG | |  |  |  |  |  |  |
| SvarPyV1 LD-PCR | 7796-s | 1 | SvarPyV1 genome | | | CCTTACCCTGTGACATCATTGC | | approx. 5000 | | 55 | | this study | |
|  | 7796-s |  |  |  |  | TGAGAGTCACATTGAAATATCTAGGT | |  |  |  |  |  |  |
|  | 7797-s | 2 |  |  |  | ACCCTGTGACATCATTGCTGT | | approx. 5000 | | 55 | |  |  |
|  | 7797-as |  |  |  |  | AATCCACAAATATCAGCACATGAA | |  |  |  |  |  |  |
|  | 7798-s | 3 |  |  |  | CCTCATGCCCAAAATGCAGG | | approx. 5000 | | 55 | |  |  |
|  | 7798-as |  |  |  |  | TGAAAGAAAGAGCCCATCTCC | |  |  |  |  |  |  |
| **Herpesvirus** |  |  |  | |  | | |  | |  | |  | |
| Generic herpesvirus DPOL PCR | 285-s DFA | 1 | ORF 09 DPOL CDS | | GAYTTYGC(N/I)AGYYT(N/I)TAYCC (DFA) | | | approx. 700 | | 46 | | (46) | |
|  | 285-s ILK |  |  |  | TCCTGGACAAGCAGCAR(N/I)YSGC(N/I)MT(N/I)AA (ILK) | | | approx. 500 | |  |  |  |  |
|  | 285-as KG1 |  |  |  | GTCTTGCTCACCAG(N/I)TC(N/I)AC(N/I)CCYTT (KG1) | | |  | |  |  |  |  |
|  | 286-s TGV | 2 |  |  | TGTACCTCGGTGTAYGG(N/I)TTYAC(N/I)GG(N/I)GT (TGV) | | | approx. 210-240 | | 46 | |  |  |
|  | 286-as IYG |  |  |  | CACAGAGTCCGTRTC(N/I)CCRTA(N/I)AT (IYG) | | |  | |  |  |  |  |
| Generic betaherpesvirus gB PCR | 2743-s EAW | 1 | ORF 08 gB CDS | | CGCAAATCGCAGA(N/I)KC(N/I)TGGTG (2743s EAW) | | | 320 | | 46 | | (52) | |
|  | 2746-as NEI |  |  |  | TGGTTGCCCAACAG(N/I)ATYTCRTT (2746as NEI) | | |  |  |  |  |  |  |
|  | 2744-s KIN | 2 |  |  | TTCAAGGAACTCAGYAARAT(N/I)AAYCC (2744s KIN) | | | 250 | | 46 | |  |  |
|  | 2745-as YGQ |  |  |  | CGTTGTCCTC(N/I)CC(N/I)ARYTG(N/I)CC (2745as YGQ) | | |  |  |  |  |  |  |
|  |  |  |  | |  | | |  | |  | |  | |
|  |  |  |  | |  | | |  | |  | |  | |
| Generic gammaherpesvirus gB PCR | 2759-s AYD | 1 | ORF 08 gB CDS | | CCTCCCAGGTTCARTWYGCMTAYGA | | | 700 | | 46 | | (51) | |
|  | 2762-as EYN |  |  |  | CCGTTGAGGTTCTGAGTGTARTARTTRTAYTC | | |  |  |  |  |  |  |
|  | 2760-s SVM | 2 |  |  | AAGATCAACCCCAC(N/I)AG(N/I)GT(N/I)ATG | | | 500 | | 46 | |  |  |
|  | 2761-as ETM |  |  |  | GTGTAGTAGTTGTACTCCCTRAACAT(N/I)GTYTC | | |  |  |  |  |  |  |
| SvulBHV1 gB-DPOL LD-PCR | 7690-s | 1 | SvulBHV1 gB-DPOL CDS | | GATCGTCCCGTGTCTGCTAG | | | approx. 3000 | | 55 | | this study | |
|  | 7690-as |  |  |  | ACGAACATCCAACGCCGAAG | | |  |  |  |  |  |  |
|  | 7691-s | 2 |  |  | GGGCGATGTCATTGCATTGG | | | approx. 3000 | | 55 | |  |  |
|  | 7691-as |  |  |  | ATCATCATCCGAGATCCCGA | | |  |  |  |  |  |  |
|  | 7692-s | 3 |  |  | TTGCATTGGCCGAATGTGTC | | | approx. 3000 | | 55 | |  |  |
|  | 7692-as |  |  |  | CGAGATCCCGAATTGTTGCG | | |  |  |  |  |  |  |
| ScarBHV1 specific nested DPOL PCR | 7815-s | 1 | ScarBHV1 DPOL CDS | | CACAATCTGTGTTATTCCACGTT | | | 391 | | 59 | | this study | |
|  | 7815-as |  |  |  | CGACATCGGCAAAATGTCCG | | |  |  |  |  |  |  |
|  | 7816-s | 2 |  |  | TTCCACGTTAGTTCCGGACG | | | 352 | | 60 | |  |  |
|  | 7816-as |  |  |  | GTAATTCGAGGTCGCCACCA | | |  |  |  |  |  |  |
| ScarBHV1 heminested DPOL PCR | 285-s DFA | 1 | ScarBHV1 DPOL CDS | | GAYTTYGC(N/I)AGYYT(N/I)TAYCC (DFA) | | | approx. 390 | | 46 | | this study | |
|  | 7776-as |  |  |  | CCACCAACATATCCCTCCCG | | |  | |  | |  |  |
|  | 285-s DFA | 2 |  |  | GAYTTYGC(N/I)AGYYT(N/I)TAYCC (DFA) | | | approx. 350 | | 46 | |  |  |
|  | 7777-as |  |  |  | GATCGCGACACACGGCAA | | |  | |  | |  |  |
| ScarGHV1 gB-DPOL LD-PCR | 7666-s | 1 | ScarGHV1 gB-DPOL CDS | GTATGACCAGCCCGTCTCTG | | | approx. 3400 | | | 60 | this study | | |
|  | 7666-as |  |  | TGTTCTAATTGGCTCCGCGT | | |  |  |  |  |  |  |  |
|  | 7667-s | 2 |  | TCTGCTAAGAGACTCGGGGA | | | approx. 3400 | | | 59 |  |  |  |
|  | 7667-as |  |  | TCCGCGTGACTGATTCAACA | | |  |  |  |  |  |  |  |
| ScarGHV1 specific nested DPOL PCR | 7666-s | 1 | ScarGHV1 DPOL CDS | GTATGACCAGCCCGTCTCTG | | | 436 | | | 58 | this study | | |
|  | 7668-as |  |  | AACATTGGAAAGCTTTTTCTCTGA | | |  | | |  |  |  |  |
|  | 7667-s | 2 |  | TCTGCTAAGAGACTCGGGGA | | | 413 | | | 58 |  |  |  |
|  | 7669-as |  |  | GGAAAGCTTTTTCTCTGATTTGCT | | |  | | |  |  |  |  |
| ScarGHV2 heminested DPOL PCR | 285-s DFA | 1 | ScarGHV2 DPOL CDS | GAYTTYGC(N/I)AGYYT(N/I)TAYCC (DFA) | | | approx. 390 | | | 46 | this study | | |
|  | 7778-as |  |  | AGCTCCTCAACAAATTGTTTGGA | | |  | | |  |  |  |  |
|  | 285-s DFA | 2 |  | GAYTTYGC(n/i)AGYYT(N/I)TAYCC (DFA) | | | approx. 350 | | | 46 |  |  |  |
|  | 7779-as |  |  | CCTCAACAAATTGTTTGGATTTTTCCA | | |  | | |  |  |  |  |
| UricGHV1 gB-DPOL LD-PCR | 7772-s | 1 | UricGHV1 gB-DPOL CDS | CAAGGCCGCAAGTCACATTC | | | approx. 3500 | | | 60 | this study | | |
|  | 7772-as |  |  | GGGTTCGGGGTGAATGGTAG | | |  |  |  |  |  |  |  |
|  | 7773-s | 2 |  | GCCGCAAGTCACATTCAAAT | | | approx. 3500 | | | 58 |  |  |  |
|  | 7773-as |  |  | GTAGACCCAAGGTGGCCTG | | |  |  |  |  |  |  |  |
| UricGHV1 specific nested gB PCR | 7772-s | 1 | UricGHV1 gB CDS | CAAGGCCGCAAGTCACATTC | | | 532 | | | 60 | this study | | |
|  | 7811-as |  |  | GGATGCCTCCCAGTGGATTT | | |  | | |  |  |  |  |
|  | 7773-s | 2 |  | GCCGCAAGTCACATTCAAAT | | | 520 | | | 58 |  |  |  |
|  | 7812-as |  |  | ACTGATGAATCCACTCACTATTGA | | |  | | |  |  |  |  |
| TstrGHV1 specific nested DPOL PCR | 7813-s | 1 | TstrGHV1 DPOL CDS | ATCCAGGCTCATAACTTATGCT | | | 464 | | | 59 | this study | | |
|  | 7813-as |  |  | ACTTTAAATCTAGCTGTAGGATTTGGA | | |  |  |  |  |  |  |  |
|  | 7814-s | 2 |  | CCCTAACCTAAAACCTGATGATTATGA | | | 397 | | | 60 |  |  |  |
|  | 7814-as |  |  | TCTAGCTGTAGGATTTGGAATGAA | | |  |  |  |  |  |  |  |
| TstrGHV1 heminested DPOL PCR | 285-s DFA | 1 | TstrGHV1 DPOL CDS | GAYTTYGC(N/I)AGYYT(N/I)TAYCC | | | 400 | | | 46 | this study | | |
|  | 7780-as |  |  | GGTGAGATCCTCAACAAATTGTT | | |  | | |  |  |  |  |
|  | 285-s DFA | 2 |  | GAYTTYGC(N/I)AGYYT(N/I)TAYCC | | | 380 | | | 46 |  |  |  |
|  | 7781-as |  |  | TGTTTAGATTTTTCCAGCATAGACC | | |  |  |  |  |  |  |  |
| CeryBHV1 specific nested DPOL PCR | 7819-s | 1 | CeryBHV1 DPOL CDS | GCACACAATCTATGTTACTCGACG | | | 461 | | | 61 | this study | | |
|  | 7819-as |  |  | CGAACATCCAAATCAGACGTCG | | |  |  |  |  |  |  |  |
|  | 7820-s | 2 |  | GACGTTGGTTCTGGGAGGAG | | | 415 | | | 61 |  |  |  |
|  | 7820-as |  |  | CACGTCTTCCGACATCCCAA | | |  |  |  |  |  |  |  |
| CeryBHV1 heminested DPOL PCR | 285-s DFA | 1 | CeryBHV1 DPOL CDS | GAYTTYGC(N/I)AGYYT(N/I)TAYCC | | | 390 | | | 46 | this study | | |
|  | 7774-as |  |  | TGACCAACATCTCCCTTCCG | | |  | | |  |  |  |  |
|  | 285-s DFA | 2 |  | GAYTTYGC(N/I)AGYYT(N/I)TAYCC | | | 350 | | | 46 |  |  |  |
|  | 7775-as |  |  | ATGGCGAGACAGGGCAAC | | |  |  |  |  |  |  |  |
| CeryGHV1 specific nested DPOL PCR | 7817-s | 1 | CeryGHV1 DPOL CDS | GAGCATAATTCAAGCACATAACTTATG | | | 466 | | | 60 | this study | | |
|  | 7817-as |  |  | AAAGTAGGACTCTGTGTTACACTT | | |  |  |  |  |  |  |  |
|  | 7818-s | 2 |  | ACGAGTAATAATCTACACAAGTTTTCT | | | 418 | | | 59 |  |  |  |
|  | 7818-as |  |  | AGGACTCTGTGTTACACTTAACTT | | |  |  |  |  |  |  |  |
| CeryGHV1 heminested DPOL PCR | 285-s DFA | 1 | CeryGHV1 DPOL CDS | GAYTTYGC(N/I)AGYYT(N/I)TAYCC | | | 415 | | | 46 | this study | | |
|  | 7782-as |  |  | ACAAACAACTTTGTAATTTCCAACAT | | |  | | |  |  |  |  |
|  | 285-s DFA | 2 |  | GAYTTYGC(N/I)AGYYT(N/I)TAYCC | | | 360 | | | 46 |  |  |  |
|  | 7783-as |  |  | GGCGATATTCAGACATGGCA | | |  | | |  |  |  |  |
|  |  |  |  |  | | |  | | |  |  | | |
|  |  |  |  |  | | |  | | |  |  | | |
|  |  |  |  |  | | |  | | |  |  | | |
| CpreBHV1 specific nested DPOL PCR | 7849-s | 1 | CpreBHV1 DPOL CDS | AATTATGGCGCACAACCTGT | | | 449 | | | 56 | this study | | |
|  | 7849-as |  |  | CGAAAACCGCATCTTCTGGC | | |  |  |  |  |  |  |  |
|  | 7850-s | 2 |  | ACGTTGATTCGCGATGGAGA | | | 408 | | | 57 |  |  |  |
|  | 7850-as |  |  | TTCTGGCACCCCAATGTGTT | | |  |  |  |  |  |  |  |
| CpreBHV1 heminested DPOL PCR | 285-s DFA | 1 | CpreBHV1 DPOL CDS | GAYTTYGC(N/I)AGYYT(N/I)TAYCC | | | 500 | | | 46 | this study | | |
|  | 7845-as |  |  | TTCTGGCACCCCAATGTGTT | | |  |  |  |  |  |  |  |
|  | 285-s DFA | 2 |  | GAYTTYGC(N/I)AGYYT(N/I)TAYCC | | | 500 | | | 46 |  |  |  |
|  | 7846-as |  |  | GCCACCAACATATCCCTCCC | | |  |  |  |  |  |  |  |
| CpreGHV1 heminested DPOL PCR | 285-s DFA | 1 | CpreGHV1 DPOL CDS | GAYTTYGC(N/I)AGYYT(N/I)TAYCC | | | 500 | | | 46 | this study | | |
|  | 7847-as |  |  | CCAACATTTTACGCCCCTGTG | | |  |  |  |  |  |  |  |
|  | 285-s DFA | 2 |  | GAYTTYGC(N/I)AGYYT(N/I)TAYCC | | | 500 | | | 46 |  |  |  |
|  | 7848-as |  |  | ACGCCCCTGTGAGGTTATTG | | |  |  |  |  |  |  |  |
| Mycoplasma-PCR | 634-s |  | ribosomal RNA | GGGAGCAAACAGGATTAGATACCCT | | | 280 | | | 55 | (53) | | |
|  | 634-as |  |  | TGCACCATCTGTCACTCTGTTAACCTC | | |  |  |  |  |  |  |  |

^a^ I = Inosine; R/I = A or G or I, in the same ratio

^b^ N/I = A or C or G or T or I, in the same ratio

^c^ LD-PCR = long-distance PCR
