# Supplementary figures and images for: Search for polyoma-, herpes-, and bornaviruses in squirrels of the family Sciuridae
Source: Virol J. 2020 Mar 27;17:42. doi: 10.1186/s12985-020-01310-4 (PMC7099801; doi:10.1186/s12985-020-01310-4)

## Slide 1
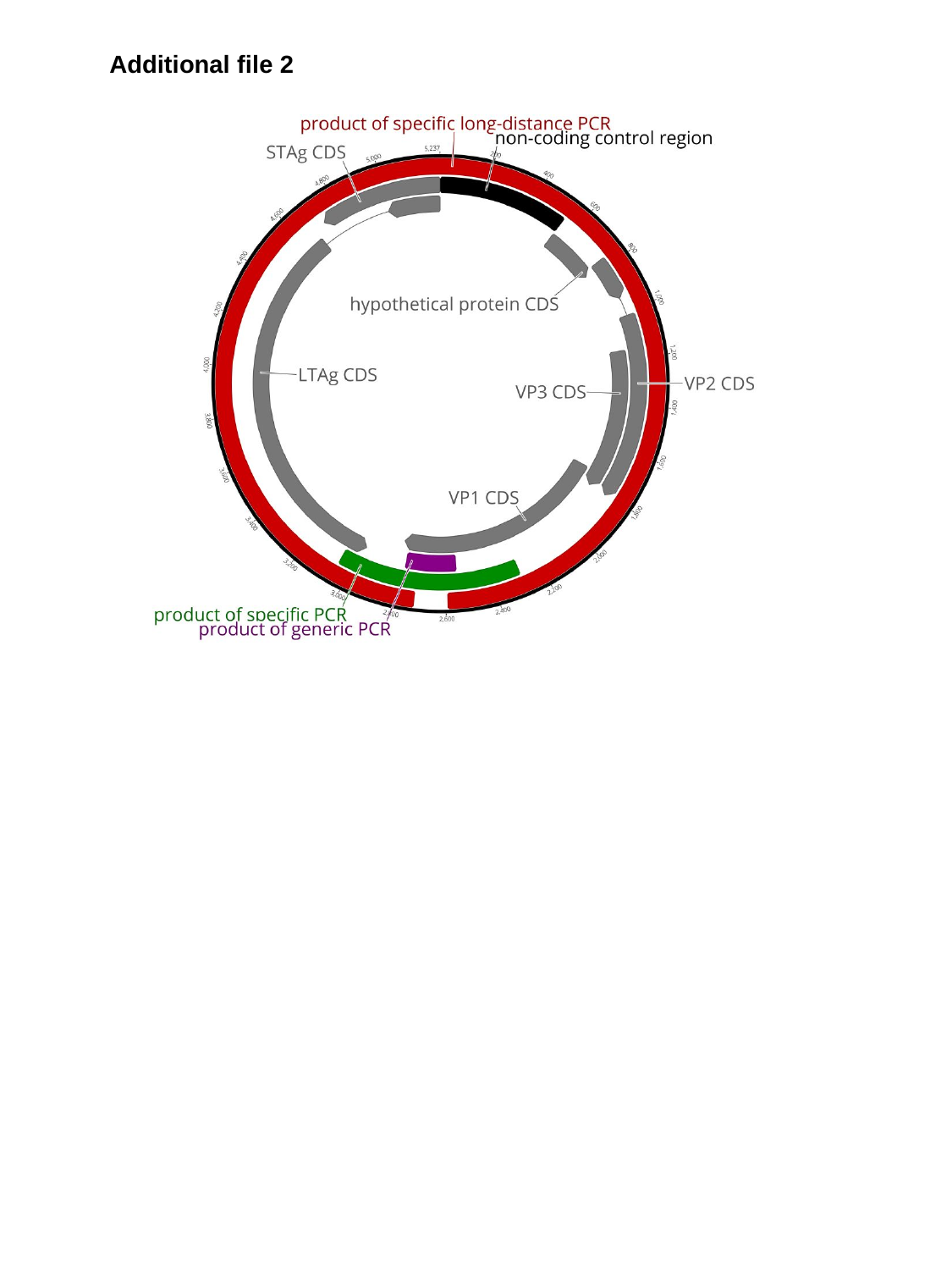

Additional file 2

Supplement: Supplementary file 2 — Additional file 2. Flow chart of multi-level PCR analysis for detection of squirrel polyomaviruses. Generic nested VP1 PCR (second-round product displayed as magenta-coloured bar) with degenerate primers was conducted. For full genome amplification, this was followed by specific nested long-distance PCR (LD-PCR; second-round product of approximately 5 kbp shown as red bar) and overlapping standard nested PCR (second-round product of approximately 800 bp shown as green bar) with specific primers. Grey bars represent coding sequences, black bar the non-coding control region. [file 12985_2020_1310_MOESM2_ESM.pptx]

## Slide 1
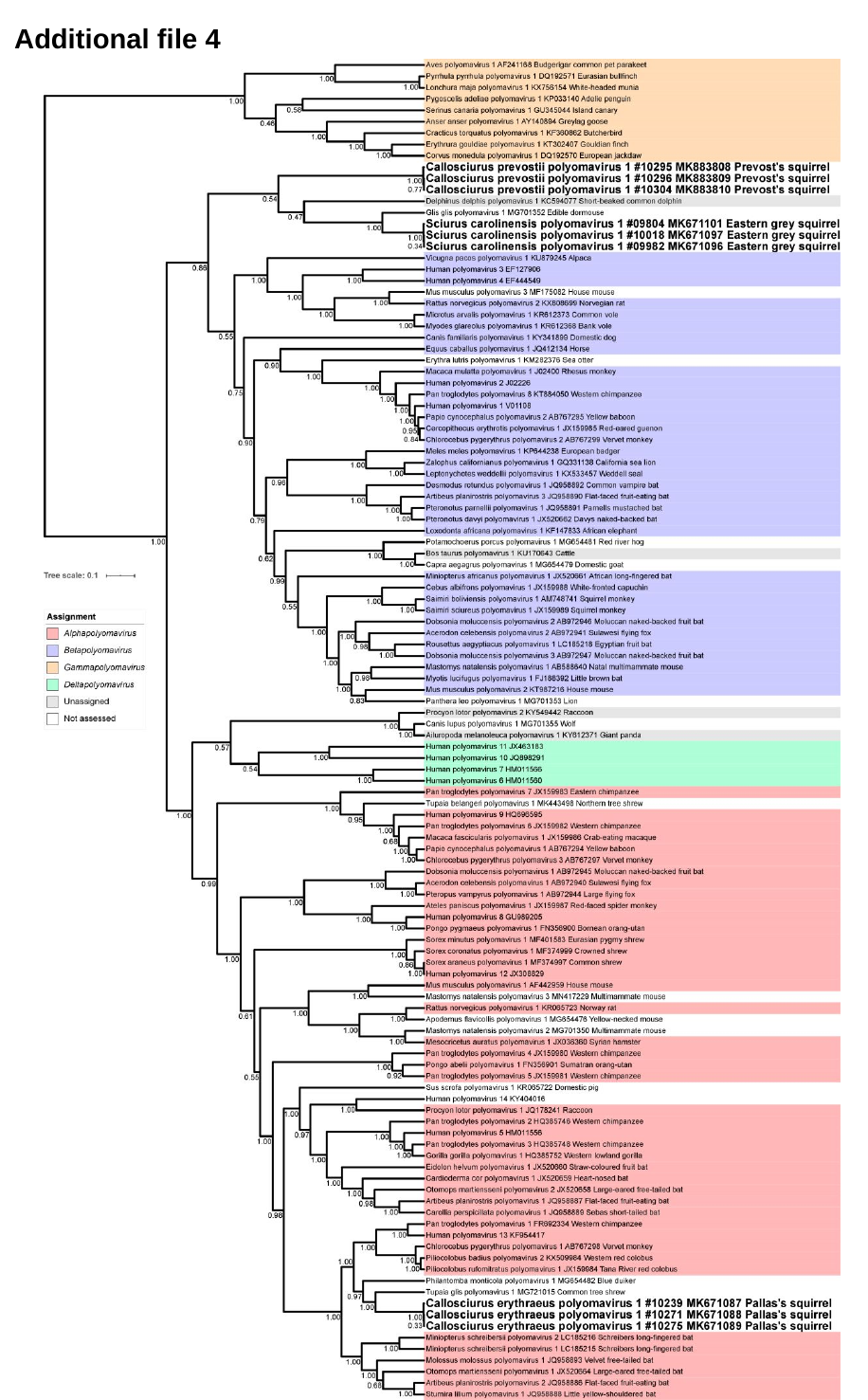

Additional file 4

Supplement: Supplementary file 4 — Additional file 4. Maximum clade credibility tree analysis of polyomaviruses based on conserved amino acid blocks of the LTAg sequences. Phylogenetic relationships of polyomaviruses, including classification of the novel viruses, based on conserved amino acid blocks of LTAg sequence. Branch support values displayed at the nodes correspond to their posterior probability. For further details see legend of Fig. 3. [file 12985_2020_1310_MOESM4_ESM.pptx]

## Slide 1
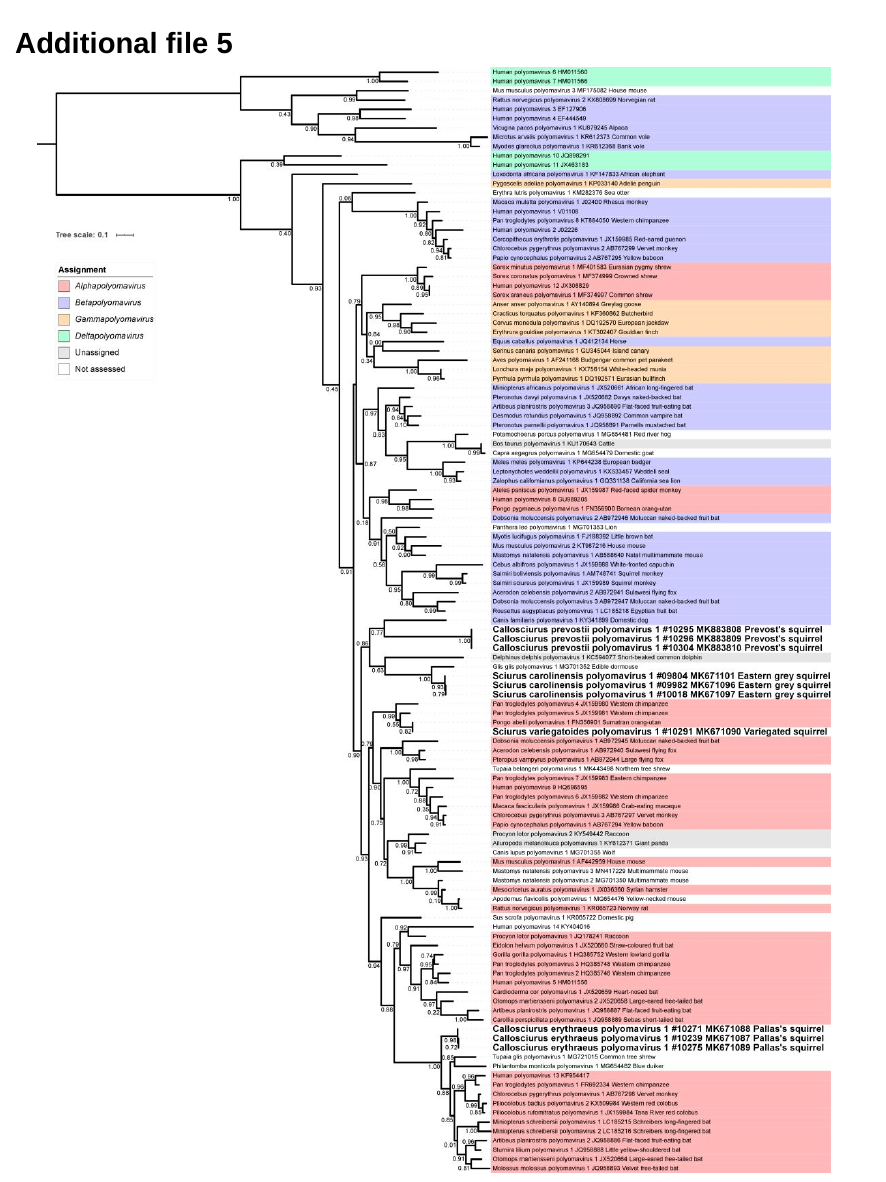

Additional file 5

Supplement: Supplementary file 5 — Additional file 5. Maximum likelihood tree analysis of polyomaviruses based on conserved amino acid blocks of the VP1 sequences. Phylogenetic relationships of polyomaviruses, including classification of the novel viruses, based on conserved amino acid blocks of VP1 sequence. Branch support values displayed at the nodes were assessed using Shimodaira-Hasagawa-like approximate likelihood ratio tests (SH-like aLRT). For further details see legend of Fig. 3. [file 12985_2020_1310_MOESM5_ESM.pptx]

## Slide 1
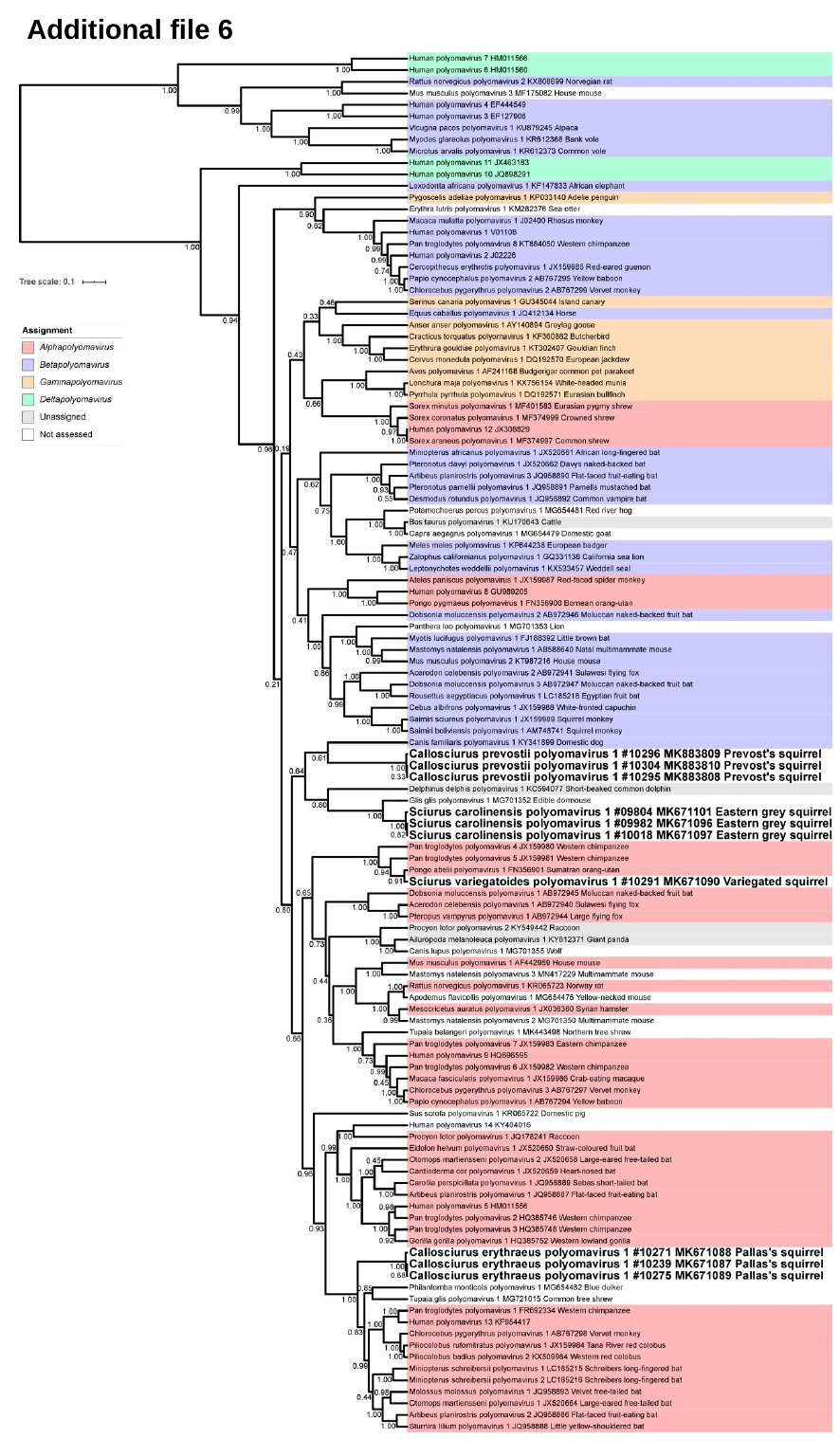

Additional file 6

Supplement: Supplementary file 6 — Additional file 6. Maximum clade credibility tree analysis of polyomaviruses based on conserved amino acid blocks of the VP1 sequences. Phylogenetic relationships of polyomaviruses, including classification of the novel viruses, based on conserved amino acid blocks of VP1 sequence. Branch support values displayed at the nodes correspond to their posterior probability. For further details see legend of Fig. 3. [file 12985_2020_1310_MOESM6_ESM.pptx]

## Slide 1
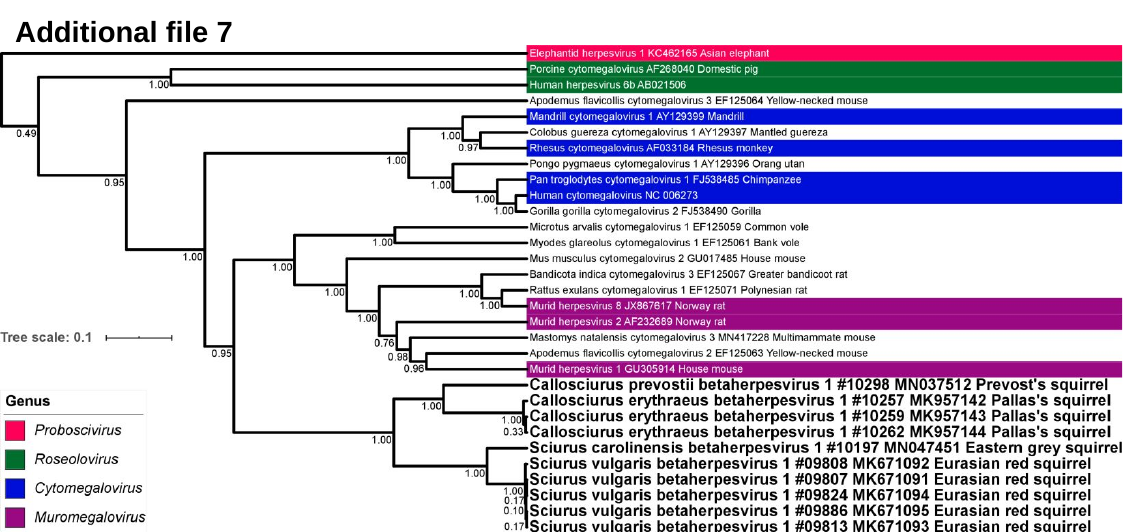

Additional file 7

Supplement: Supplementary file 7 — Additional file 7. Maximum clade credibility tree analysis of betaherpesviruses based on conserved amino acid blocks of the DPOL sequences. Phylogenetic relationships of betaherpesviruses, including classification of the novel viruses, based on conserved amino acid blocks of DPOL sequence. Branch support values displayed at the nodes correspond to their posterior probability. For further explanation see legend of Fig. 4. [file 12985_2020_1310_MOESM7_ESM.pptx]

## Slide 1
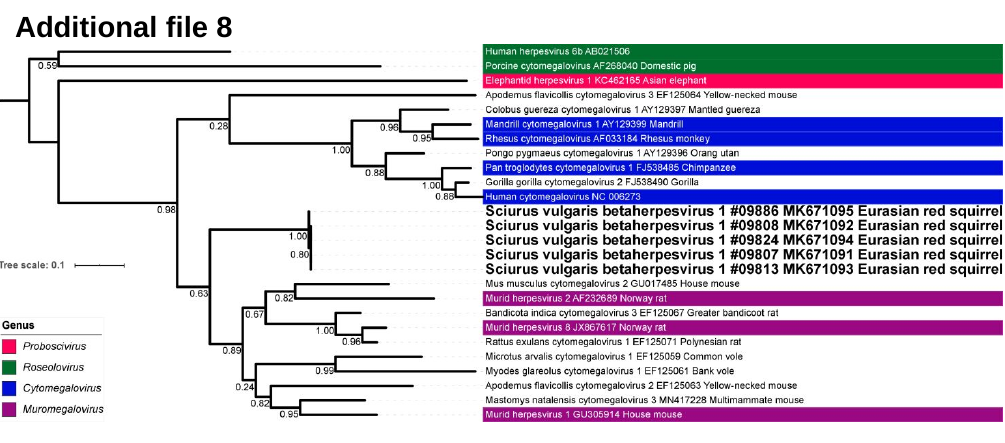

Additional file 8

Supplement: Supplementary file 8 — Additional file 8. Maximum likelihood tree analysis of betaherpesviruses based on conserved amino acid blocks of the gB sequences. Phylogenetic relationships of betaherpesviruses, including classification of the novel viruses, based on conserved amino acid blocks of gB sequence. Branch support values displayed at the nodes were assessed using Shimodaira-Hasagawa-like approximate likelihood ratio tests (SH-like aLRT). For further explanation see legend of Fig. 4. [file 12985_2020_1310_MOESM8_ESM.pptx]

## Slide 1
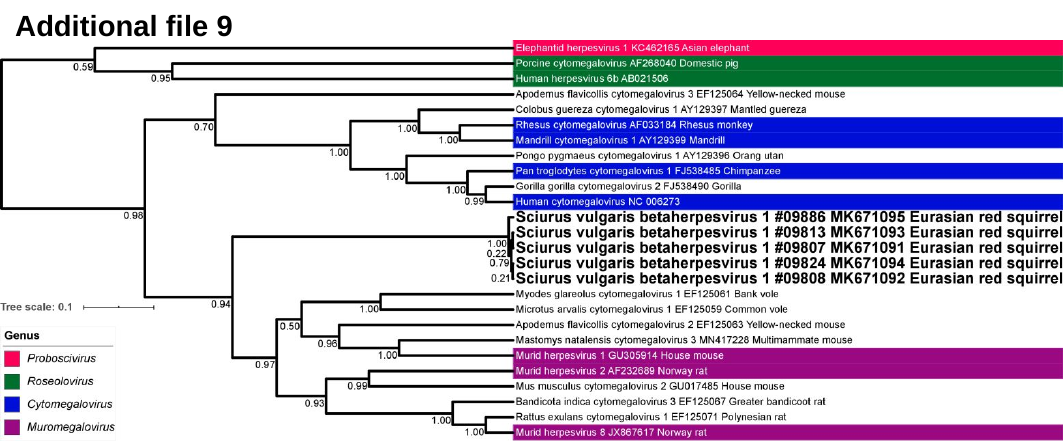

Additional file 9

Supplement: Supplementary file 9 — Additional file 9. Maximum clade credibility tree analysis of betaherpesviruses based on conserved amino acid blocks of the gB sequences. Phylogenetic relationships of betaherpesviruses, including classification of the novel viruses, based on conserved amino acid blocks of gB sequence. Branch support values displayed at the nodes correspond to their posterior probability. For further explanation see legend of Fig. 4. [file 12985_2020_1310_MOESM9_ESM.pptx]

## Slide 1
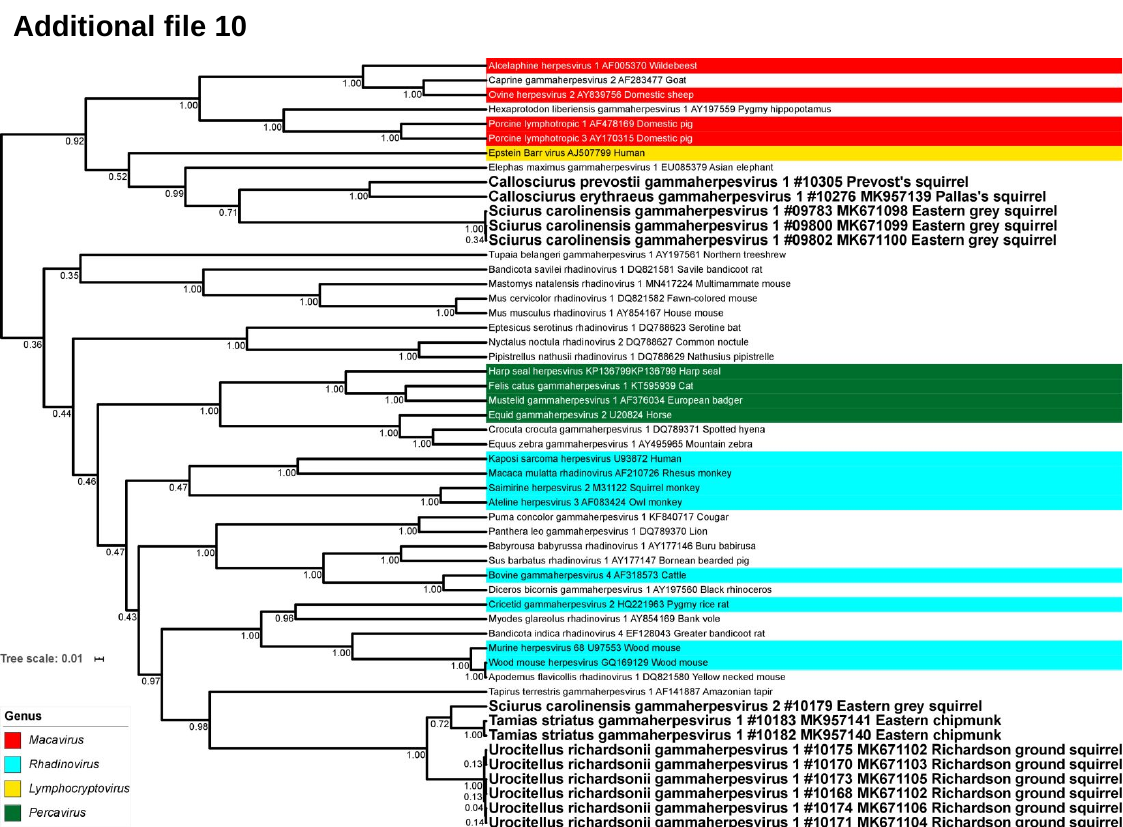

Additional file 10

Supplement: Supplementary file 10 — Additional file 10. Maximum clade credibility tree analysis of gammaherpesviruses based on conserved amino acid blocks of the DPOL sequences. Phylogenetic relationships of gammaherpesviruses, including classification of the novel viruses, based on conserved amino acid blocks of DPOL sequence. Branch support values displayed at the nodes correspond to their posterior probability. For further explanation see legend of Fig. 5. [file 12985_2020_1310_MOESM10_ESM.pptx]

## Slide 1
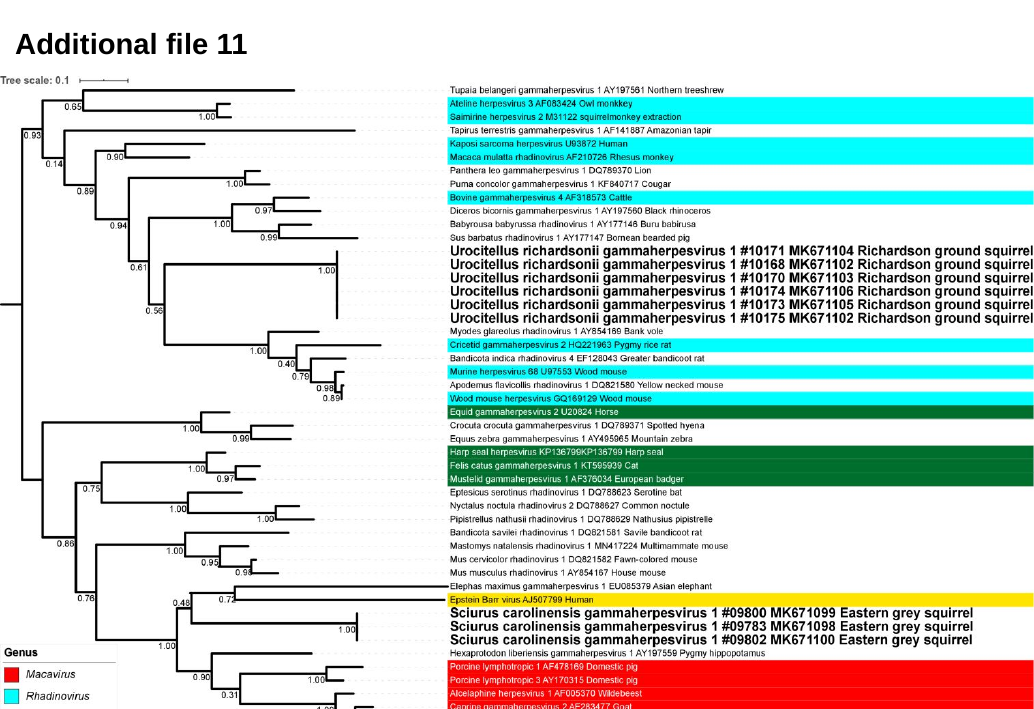

Additional file 11

Supplement: Supplementary file 11 — Additional file 11. Maximum likelihood tree analysis of gammaherpesviruses based on conserved amino acid blocks of the gB sequences. Phylogenetic relationships of gammaherpesviruses, including classification of the novel viruses, based on conserved amino acid blocks of gB sequence. Branch support values displayed at the nodes were assessed using Shimodaira-Hasagawa-like approximate likelihood ratio tests (SH-like aLRT). For further explanation see legend of Fig. 5. [file 12985_2020_1310_MOESM11_ESM.pptx]

## Slide 1
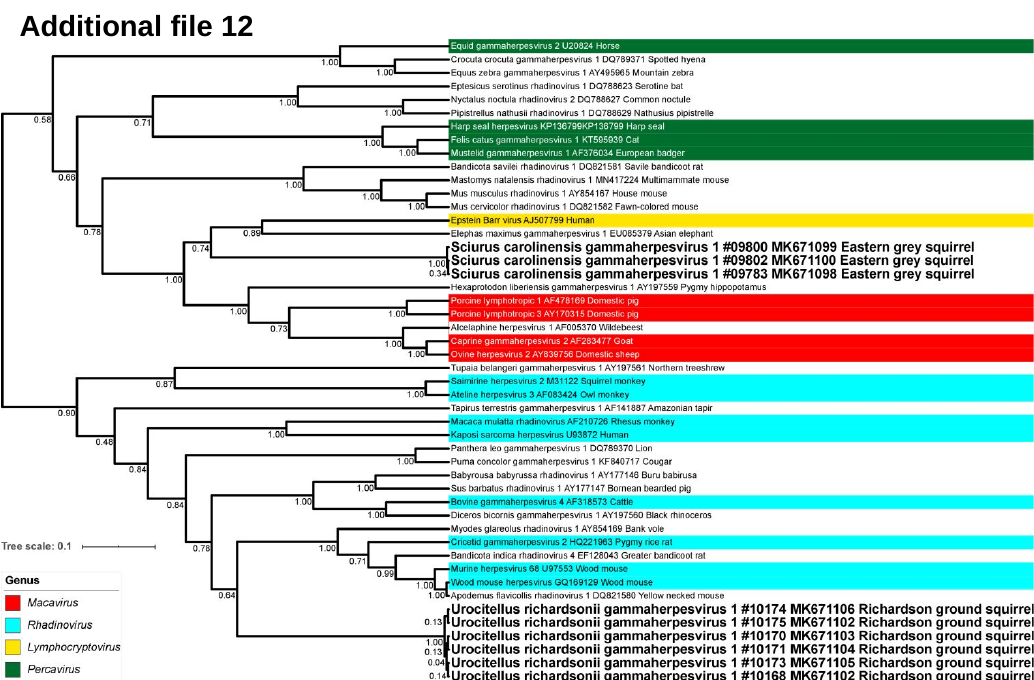

Additional file 12

Supplement: Supplementary file 12 — Additional file 12. Maximum clade credibility tree analysis of gammaherpesviruses based on conserved amino acid blocks of the gB sequences. Phylogenetic relationships of gammaherpesviruses, including classification of the novel viruses, based on conserved amino acid blocks of gB sequence. Branch support values displayed at the nodes correspond to their posterior probability. For further explanation see legend of Fig. 5. [file 12985_2020_1310_MOESM12_ESM.pptx]
