# Supplementary material for: Search for polyoma-, herpes-, and bornaviruses in squirrels of the family Sciuridae
Source: Virol J. 2020 Mar 27;17:42. doi: 10.1186/s12985-020-01310-4 (PMC7099801; doi:10.1186/s12985-020-01310-4)
Supplement: Supplementary file 3 — Additional file 3. Flow chart of multi-level PCR analysis for detection of squirrel herpesviruses. Generic nested DPOL PCR (product: bar in magenta) with degenerate primers was carried out. For extended sequence determination, this was followed by generic gB PCR (blue) with degenerate primers and subsequent long-distance PCR (LD-PCR) (red) with specific primers. Products of the second PCR rounds are shown. The sequences of the generic DPOL PCR product and the extended DPOL PCR product build a contiguous sequence of 0.4–0.5 kbp (black). The sequences of the generic gB and the generic DPOL PCR product build together with the LD-PCR-derived sequence a contiguous sequence of approximately 3.3 kbp (black). On top of the figure, coding sequences are displayed by grey bars. The arrow heads indicate the direction of transcription. [file 12985_2020_1310_MOESM3_ESM.pptx]

## Slide 1
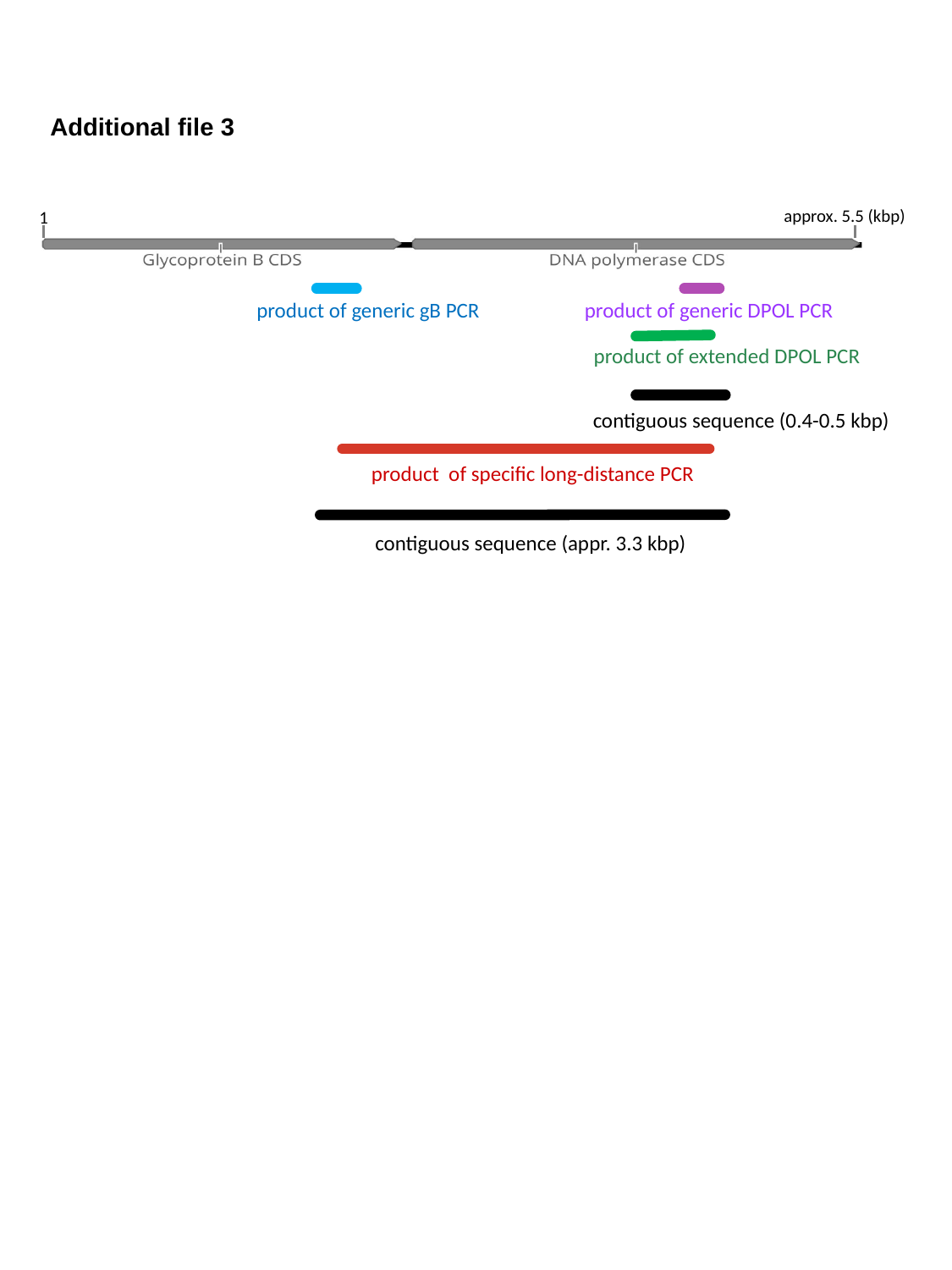

Additional file 3
approx. 5.5 (kbp)
1
product of generic gB PCR
product of generic DPOL PCR
product of extended DPOL PCR
contiguous sequence (0.4-0.5 kbp)
product of specific long-distance PCR
contiguous sequence (appr. 3.3 kbp)
